# Combinatorial Screening of Cationic Lipidoids Reveals How Molecular Conformation Affects Membrane-Targeting Antimicrobial Activity

**DOI:** 10.1021/acsami.3c05481

**Published:** 2023-08-21

**Authors:** James Jennings, Dunja Ašćerić, Enrico Federico Semeraro, Karl Lohner, Nermina Malanovic, Georg Pabst

**Affiliations:** † Institute of Molecular Biosciences, University of Graz, NAWI Graz, 8010 Graz, Austria; ‡ Field of Excellence BioHealth, University of Graz, 8010 Graz, Austria

**Keywords:** membrane-active compounds, quaternary ammonium compounds, combinatorial synthesis, lipidoids, small-angle X-ray scattering

## Abstract

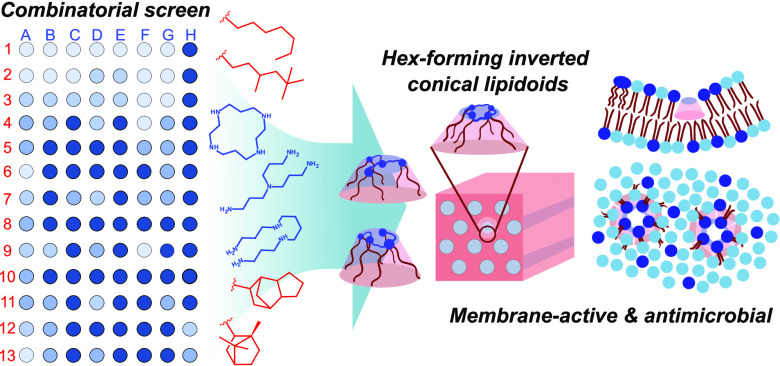

The search for next-generation antibacterial compounds that overcome the development of resistance can be facilitated by identifying how to target the cell membrane of bacteria. Understanding the key molecular features that enable interactions with lipids and lead to membrane disruption is therefore crucial. Here, we employ a library of lipid-like compounds (lipidoids) comprising modular structures with tunable hydrophobic and hydrophilic architecture to shed light on how the chemical functionality and molecular shape of synthetic amphiphilic compounds determine their activity against bacterial membranes. Synthesized from combinations of 8 different polyamines as headgroups and 13 acrylates as tails, 104 different lipidoids are tested for activity against a model Gram-positive bacterial strain (*Bacillus subtilis*). Results from the combinatorial screening assay show that lipidoids with the most potent antimicrobial properties (down to 2 μM) have intermediate tail hydrophobicity (i.e., c log *P* values between 3 and 4) and lower headgroup charge density (i.e., longer spacers between charged amines). However, the most important factor appeared to be the ability of a lipidoid to self-assemble into an inverse hexagonal liquid crystalline phase, as observed by small-angle X-ray scattering (SAXS) analysis. The lipidoids active at lowest concentrations, which induced the most significant membrane damage during propidium iodide (PI) permeabilization assays, were those that aggregated into highly curved inverse hexagonal liquid crystal phases. These observations suggest that the introduction of strong curvature stress into the membrane is one way to maximize membrane disruption and lipidoid antimicrobial activity. Lipidoids that demonstrated the ability to furnish this phase consisted of either (i) branched or linear headgroups with shorter linear tails or (ii) cyclic headgroups with 4 bulky nonlinear tails. On the contrary, lipidoids previously observed to adopt disc-like conformations that pack into bicontinuous cubic phases were significantly less effective against *B. subtilis*. The discovery of these structure–property relationships demonstrates that it is not simply a balance of hydrophobic and hydrophilic moieties that govern membrane-active antibacterial activity, but also their intrinsic curvature and collective behavior.

## Introduction

Antimicrobial resistance in bacteria is already a public health crisis, as several common drugs are now ineffective against many species of pathogenic bacteria. The need for next-generation antimicrobials that preclude the development of resistance is highly pressing in light of recent reports by the World Health Organization.^1,2^ Strategically, new classes of antibiotics targeting irreversible and unspecific disruption of the protective cellular envelope of bacteria, i.e., the cytoplasmic membrane, are far less prone to resistance than those that specifically target active sites, such as enzymes.^3^ Such membrane-active compounds can broadly be divided into two categories: antimicrobial peptides (AMPs),^4−6^ some of which form part of innate immune systems of species ranging from microorganisms to mammals,^7^ and synthetic quaternary ammonium compounds (QACs).^8^ Although the chemical structures of these two classes of molecules are distinct, both possess common features that are considered to be key to their membrane-disruptive mode of action: multiple cationic moieties and hydrophobic groups. These characteristics allow such compounds to impart a combination of unspecific physical interactions with a cell membrane, dominated by electrostatic interactions with lipid headgroups and hydrophobic interactions with hydrocarbons of the membrane interior, which balance with the system’s translational and configurational entropy.

Designing highly effective membrane-active compounds necessitates a specification of the class of bacteria being targeted, which differ significantly in the composition of their cellular envelope and hence their collective physicochemical properties.^9^ Gram-negative bacteria, such as *Escherichia coli*, comprise an outer and inner (cytoplasmic) membrane, with a peptidoglycan layer in the periplasmic space, while Gram-positive bacteria only contain a cytoplasmic membrane and surrounding (thicker) layer of peptidoglycan as the cell wall. The presence of an additional outer membrane in Gram-negative bacteria typically results in a lower susceptibility toward many antibiotics. In both cases, the cytoplasmic membrane is rich in anionic lipids, particularly cardiolipin and phosphatidylglycerol lipids.^10^ Thus, compounds containing positively charged moieties tend to be employed to enhance electrostatic attraction to bacterial membranes.^11^ The need for a hydrophobic component is crucial to enabling partitioning into the membrane’s interior, and governs cell membrane disruption once inserted into the membrane. The apolar volume of membrane-active compounds is mostly responsible for inducing hydrocarbon splay within the lipid bilayer and thus the probability for the formation of membrane defects or pores. These interactions can lead to cell death either via lysis of cytosolic material or by facilitating the translocation of the compounds into the cell to cause cell death by another mechanism.^12^

There is a particular dearth of understanding about the balance of molecular features that govern interactions with lipid membranes from different species, thus posing severe challenges for bottom-up rational designs of membrane-active compounds. Most membrane-active compounds are active against membranes from several species (i.e., prokaryotic and eukaryotic cells) and can therefore induce significant toxic side effects as a result of host cell membrane disruption.^13^ Reducing side effects and achieving selectivity for particular pathogenic bacteria is a major goal for next-generation antimicrobials. In view of the complexity of molecular interactions that need to be balanced, combinatorial techniques are valuable in exploring structure–property relationships more rapidly than “one factor at a time” scientific approaches. Although such high-throughput screening of AMP structures has been achieved,^5,6^ studies into synthetic amphiphiles, such as QACs, are typically limited to twin-tail “gemini” surfactants, consisting of two ammonium headgroups and two linear hydrophobic tails.^14−17^ Octenidine (OCT) represents one of the most effective broad-spectrum gemini QACs, which is now widely used as an antiseptic.^18^ Although results from some studies hint that “multi-QACs” with >2 charged headgroups and multiple tails can outperform conventional gemini QACs,^19,20^ understanding why increasing structural complexity impacts antimicrobial activity remains underexplored. There is additional motivation to move toward systems with highly charged headgroups since QACs^21^ and AMPs with higher numbers of charged moieties (>10) have been observed to be more difficult for bacteria to develop resistance to.^11,22^

Therefore, different platforms of materials that enable modular molecular design are desired to provide valuable insights into structure–activity relationships and the subtle molecular-level driving forces for membrane disruption. In this study, we take a combinatorial approach to explore the membrane activity of synthetic lipid-like compounds (lipidoids) with polyamine headgroups and ≥4 hydrophobic tails. Such lipidoids were recently discovered to self-assemble into diverse liquid crystalline nanostructures upon protonation,^23,24^ relating to unique molecular conformations that result from having a multitude of hydrophobic tails. In particular, the propensity of some of these lipidoids to form aggregates with highly curved topologies suggests a high membrane-disrupting potential. We thus hypothesized that the amphiphilic structure of lipidoids should facilitate interaction with biological membrane lipids, and their unusual molecular self-assembly behavior could bestow the compounds with sufficient hydrophobic volume to disrupt membranes through monolayer curvature stress in a way that leads to antimicrobial properties.

In this paper, we demonstrate the antimicrobial activity of lipidoids by conducting minimum inhibitory concentration (MIC) assays against *Bacillus subtilis*, a representative Gram-positive bacterium, and PI permeabilization assays to confirm the membrane-disrupting mechanism. From these analyses, we uncover a number of compounds with antimicrobial activity at low concentrations (<5 μM) and garner structure–activity relationships unveiling the importance of tail hydrophobicity and headgroup charge density. Furthermore, using SAXS analysis, we find a strong correlation between the liquid crystalline aggregate structures formed by lipidoids with their propensity to permeabilize membranes. Finally, hemolytic assays are used to reveal some lipidoid structures with high selectivity for bacterial over mammalian cell membranes.

## Results and Discussion

### Modular Synthesis of Lipidoids

**Figure 1:**
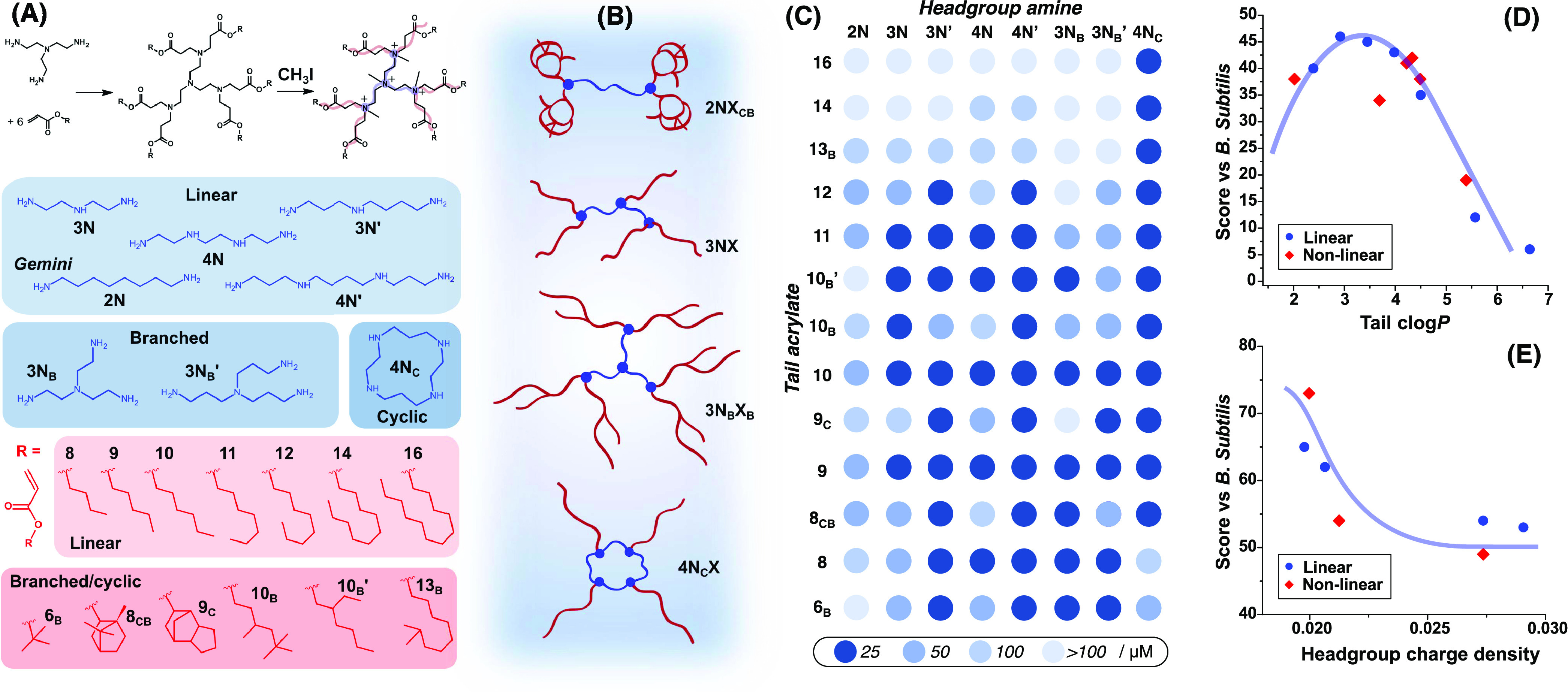
(A) Synthetic route to multitail lipidoids adopted in this study, prepared by reacting polyamines (headgroups, blue) and hydrophobic acrylates (tails, red). Selected polyamines are arranged either in a linear, branched, or ring architecture. Hydrocarbon substituents on acrylates comprised either linear or branched aliphatics, or arrangements of multiple alicyclic groups. Charge was introduced by methylation of headgroup amines using methyl iodide (CH_3_I) to improve colloidal stability. (B) Schematic representations of example lipidoids probed in this study from the different combinations of head and tail group architectures. (C) Results of combinatorial antimicrobial screening against *B. subtilis*, with each spot colored according to the lowest concentration at which lipidoids were active between 25 and 100 μM. Plots of (D) tail group c log *P* and (E) headgroup charge density values against antimicrobial activity calculated from a point-scoring system to demonstrate global structure–activity relationships. The transparent blue curves in panels (D) and (E) are drawn to guide the eye.

The synthesis of multitailed amine-based lipidoids can be achieved by reacting polyamines with hydrophobic acrylates (Figure 1A) in a one-step reaction that is high yielding and leaves minimal undesirable side products. This chemistry is amenable to combinatorial and high-throughput studies, as previously demonstrated for similar lipidoids used in gene delivery applications.^25,26^ In this study, we focused on lipidoid structures with 3 or more amines per headgroup and 4 or more hydrophobic tails, following our previously optimized synthetic procedure.^23,24^ Headgroups were selected from either linear, branched, or cyclic arrangements of amines with variable hydrocarbon spacer groups between the amines (Figure 1A). One gemini lipidoid was also prepared for comparison, using a primary diamine fully substituted with four tails. Previous studies showed that longer spacers in gemini QACs resulted in the highest antimicrobial activity;^27^ therefore, a diamine with an 8-carbon spacer was adopted.

Lipidoids are named using the convention *nN_x_M_y_*, where *n* is the number of reactive nitrogens, *x* describes the architecture of the headgroup (C = cyclic, B = branched), *M* is the tail length in terms of the number of atoms from the first acrylate methylene to the furthest carbon, and *y* describes the tail architecture (C = cyclic, B = branched, CB = cyclic and branched). In the cases where two different structures have the same code, a prime (**′**) is used to differentiate; this refers to headgroup structures with different aliphatic spacer lengths or tails with different branching arrangements. Linear headgroups with 3 or 4 amines (**3N**, **3N′**, **4N**, **4N′**) produced 5 or 6 tail species, respectively, while branched headgroups with 3 primary amines (**3N**_**B**_, **3N**_**B**_**′**) imparted 6 tails, and lipidoids with the cyclic tetraamine (**4N**_**C**_) were 4-tailed. Tail groups were furnished using acrylates with ester groups substituted with either linear (**8–16**) or a host of branched/cyclic moieties (Figure 1A). As in our prior study, the purity of lipidoids was analyzed by ^1^H NMR to confirm that the fully substituted species was the major product. NMR analysis also confirmed that unreacted acrylate was removed during purification to <2% w/w (see Figure S1).^24^

Lipidoid compounds designed for this study have a high number of hydrophobic tails (4–6) and relatively small hydrophilic headgroups and therefore were poorly soluble in a variety of aqueous solutions, including bacterial growth media. We previously found that protonation of lipidoid headgroups using strong acids resulted in the formation of liquid crystalline mesophases,^23,24^ but notably, these charged liquid crystals are difficult to disperse in aqueous medium without an emulsifier. Instead, lipidoid dispersion in aqueous media was facilitated by introducing permanent positive charges into the headgroup using methyl iodide.^25^ Methylation of lipidoid secondary amines affords ammonium groups that resemble headgroups of QACs but here we prepared a greater array of molecular shapes (e.g., Figure 1B) as compared to past studies. Following the addition of methylated lipidoids to Luria Broth (LB) growth medium (see the Experimental Section for details), stable turbid solutions were usually observed, indicating the formation of aggregates. Dynamic light scattering (DLS) analysis revealed that, in most cases, lipidoids formed aggregates in the range of 100–1000 nm in LB medium (Figure S2). Some lipidoids with shorter tail groups formed smaller particles (<100 nm), or were even undetectable by DLS, suggesting molecular solubilization.

### Combinatorial Screening of Antimicrobial Activity

Methylated samples of all 104 lipidoids were prepared at 25, 50, and 100 μM in LB medium for initial growth inhibition assays against *B. subtilis*. *B. subtilis* was chosen as a model for rod-shaped Gram-positive bacteria with well-studied membrane and cell wall structures, which also allowed direct comparison to results obtained for OCT.^18^ Relative to the double membrane envelopes of Gram-negative bacteria, this simpler model system allowed for easier rationalization of the lipidoid interactions with membranes and for more facile comparisons with single-membrane mammalian cell envelopes (vide infra). Bacterial growth was measured by following the optical density at 420–580 nm (OD_420–580_) of planktonic bacteria with an initial density of 10^6^ CFU/mL in LB medium. A compound was considered to have antibacterial effects when inhibiting the growth of >99.9% of bacteria. The inhibition of growth was evidenced by the complete lack of growth during an experiment (24 h) or when the onset of the log phase was significantly delayed (see the Experimental Section for further details and Figure S3).^28^ Results from initial antimicrobial assays against *B. subtilis* are displayed in Figure 1C, with darker spots indicating compounds active at lower concentrations. Several compounds were also tested for bactericidal activity using a Copacabana assay (see the Experimental Section for details), and bactericidal activity was observed at the same concentrations as growth inhibition.

The screening study revealed that antimicrobial activity was strongly dependent on lipidoid chemical structure. To facilitate a quantitative comparison of the high volume of data and to deconvolute the effects of different molecular features, a point-scoring system was devised. Scores were assigned based on concentrations at which lipidoids were active and whether they completely prevented bacterial growth during the experiment or only delayed the onset of the log phase. For example, compounds in which >99.9% growth was inhibited at 25 μM were awarded a score of 6. Of those active only at 50 μM, they were awarded 5 points if no growth was observed within 24 h. Where growth was still observed with 50 μM compound but with a <24 h delayed onset of the log phase, a score of 4 was given. These same criteria were applied to compounds active only at 100 μM to award 2 or 3 points. Finally, compounds that showed some delay in bacterial growth at 100 μM but below the threshold for 99.9% inhibition were awarded 1 point (see Table S2 for full description). This empirical scoring scheme provided an initial assessment of how each lipidoid structural variable contributed to antibacterial properties. To couple lipidoid activity with chemical structure, each head and tail group were assigned a range of quantitative descriptors, which were plotted against the total score for every lipidoid compound with that component. For example, each tail could be quantified by length (number of atoms end-to-end), molecular weight, or log *P*: a commonly used metric to quantify hydrophobicity of a compound based on its octanol–water partition coefficient (values were taken from a database of calculated values, so are denoted as c log *P*).^29^ Meanwhile, headgroups were described by c log *P* or the charge density, defined as the number of amines in the headgroup divided by the headgroup molecular weight (assuming each amine in the headgroup became charged following methylation).

Plotting each of these descriptors against total scores across the lipidoid series revealed several global correlations. For tail groups, activity showed a parabolic correlation to c log *P* (Figure 1D), tail length, and molecular weight (Figure S4A,B), with intermediate c log *P* values (ca. 3–4) and tail lengths (9–11) showing the highest antimicrobial activity. Notably, the tricyclic **9**_**C**_ tail group deviated most significantly from the trend, showing overall lower activity than other tails with equivalent length or c log *P*. A parabolic correlation between tail length and antimicrobial activity was also previously observed in the literature for some QACs against Gram-positive bacteria.^20,27,30^ This parabolic relationship suggests that a fine balance exists for tails, between being long enough to enable enough hydrophobic interactions that allow insertion into the bilayer, but shorter than average lipid tail lengths (14–18). In other words, these results indicate that a degree of hydrophobic mismatch relative to the unperturbed bilayer is crucial. For lipidoid headgroups, some correlation between activity and charge density (Figure 1E) or c log *P* (Figure S4C) was observed, with lower charge densities and more hydrophobic headgroups achieving the highest scores. This general trend showed that longer spacers between amines, which should impart larger headgroup size and/or increased headgroup flexibility, led to increased activity. Notably, despite having the lowest charge density, the diamine **2N** performed the worst of all headgroups (full scores listed in Table S3), indicating that structures based on polyamine headgroups can outperform conventional gemini QAC structures.

Despite observing the above general trends, the initial antibacterial screening experiments at 3 concentrations could not provide a complete picture of how the lipidoid structure affects activity. To gain deeper insight into a smaller number of lipidoids, we selected 27 candidates that had structural similarities but showed antimicrobial activity at a range of different concentrations. Specifically, lipidoids comprising headgroups of **4N′**, **3N**_**B**_**′**, or **4N**_**C**_ and tail groups between 8 and 12 atoms long (with linear and nonlinear architectures) were taken forward for self-assembly studies in growth medium, mechanistic investigation, and toxicity tests. Lipidoids with these headgroups should have the same number of charges within the headgroup when methylated, and **4N′** and **3N**_**B**_**′** lipidoids each have 6 tails. First, lipidoid antimicrobial activity was measured at lower concentrations to obtain the MICs for each compound. Specifically, the 20 lipidoids from this smaller library that showed antibacterial activity at 25 μM in the initial screen were now tested at lower concentrations (1–12.5 μM) to compare relative MIC values (Table 1).

**Table 1:** Summary of Minimum Inhibitory Concentration (MIC) Values against *B. subtilis* for the 27 Lipidoids Selected from the Initial Screen

MIC/μM	headgroup		
tail group	**4N′**	**3N_B_′**	**4N_C_**
**12**	50	50	3
**11**	6	50	3
**10**_**B**_**′**	25	50	2
**10**_**B**_	25	50	3
**10**	3	12.5	3
**9**_**C**_	12.5	25	2
**9**	2	2	12.5
**8**_**CB**_	25	25	2
**8**	2	2	100

MIC assays revealed 7 different lipidoids with MICs at 2 μM, an inhibitory concentration lower than many gemini QACs, and comparable with some higher-order QACs,^14,19,31−33^ and several AMPs against a variety of Gram-positive bacteria.^11,34^ These 7 most active compounds consisted of either combinations of **4N′** or **3N**_**B**_**′** headgroups with short linear tails (**8–9**) or the **4N**_**C**_ headgroup with nonlinear tails (**8**_**CB**_, **9**_**C**_, **10**_**B**_**′**). Meanwhile, **4N′** and **3N**_**B**_**′** lipidoids with nonlinear tails or longer tails tended to present higher MICs. Evidently, no specific headgroup or tail consistently produced lipidoids with the lowest MIC values. Thus, these 27 lipidoids presented an ideal library with which to investigate the origin of differences in antibacterial activities for structurally similar amphiphiles.

### Correlating Antimicrobial Activity with Lipidoid Self-Assembly

Despite some strong overall trends connecting the structure to antimicrobial activity (Figure 1D,E), it was also evident from MIC studies that certain combinations of structural features led to compounds that most effectively inhibit or kill bacteria. For example, most **4N**_**C**_-based lipidoids showed MIC <6 μM, apart from the species with linear tails 8–9 atoms long. Conversely, lipidoids with **4N′** and **3N**_**B**_**′** headgroups possessing linear tails 8–9 atoms long were active at concentrations up to 10 times lower than compounds with nonlinear tails of similar lengths (**8**_**CB**_, **9**_**C**_, **10**_**B**_, **10**_**B**_**′**).

The liquid crystalline phase behavior of multitailed lipidoids gives a strong indication of their molecular conformations and the preferred interfacial/spontaneous curvatures of the molecules.^23,24^ Thus, we sought to understand how the different conformations of lipidoids could relate to their antimicrobial activity. Self-assembly of the 27 different lipidoids from Table 1 was analyzed by SAXS, using the dispersions of methylated lipidoids in LB growth medium at 1 mM. This concentration is notably higher than used in MIC assays since SAXS analysis has limited resolution at the lowest active concentrations (i.e., <10 μM). However, DLS analysis shows the presence of aggregates of 100–1000 μm in size at concentrations of 2 μM (Figure S1), demonstrating very low critical aggregation concentrations of these compounds. Thus, the structures formed within the dispersed aggregates at 1 mM give a strong indication of the overall shape and spontaneous curvature of lipidoid molecules.

**Figure 2:**
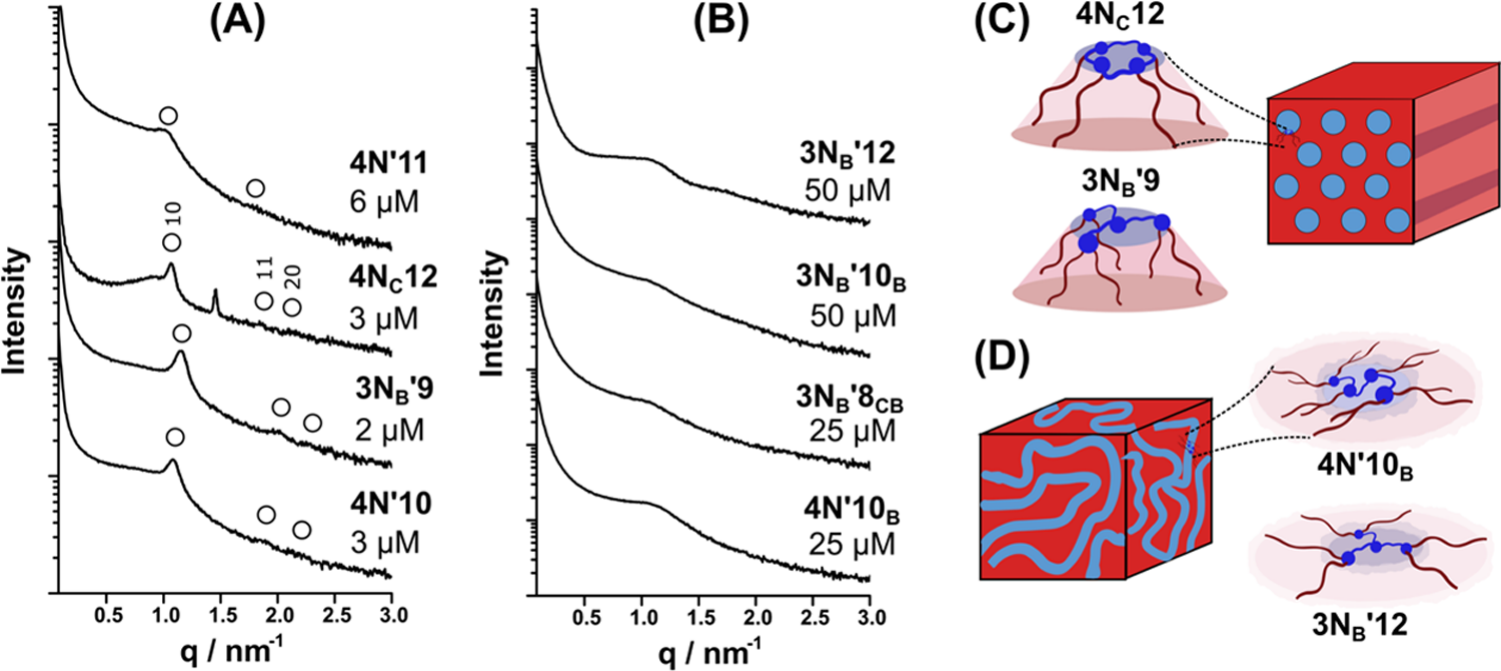
Subtracted synchrotron SAXS data collected from 1 mM lipidoid dispersions in LB medium, which displayed either (A) defined internal morphology, based on the appearance of a relatively sharp principle scattering maximum and higher-order peaks that are assignable to a hexagonal phase (open circles labeled with Miller indices), or (B) ill-defined or amorphous internal structures, identified by the presence of broad and low intensity scattering features. Each trace is labeled with the MIC value measured for the lipidoid, which demonstrates the strong correlation between the nanostructure and antimicrobial efficacy. Additional lab source SAXS patterns are presented in Figure S5. (C) Schematic of the inverse hexagonal phase comprising hydrophilic cylinders (blue) embedded in the hydrophobic volume (red), and two different representative multitailed lipidoids in inverted conical conformations. (D) Schematic of a disordered phase and putative flat disc-like conformations that may comprise it.

Results from these analyses showed two distinct behaviors for dispersed lipidoids: those which display well-defined internal liquid crystalline (LC) nanostructures (Figures 2A and S5) and those which only present disordered structures (Figures 2B and S5). Liquid crystalline structures were evidenced by the appearance of relatively narrow Bragg peak(s). In contrast, broad maxima were indicative of some fluctuations in electron density within the particles, but an ill-defined structure. Ten of the lipidoids which displayed evidence of defined internal structure within the dispersed particles showed antibacterial activity at 2 or 3 μM against *B. subtilis*. Meanwhile, the majority of lipidoids that displayed only broad scattering maxima or the absence of features altogether were those with MIC ≥ 25 μM. For samples showing sharp scattering features, additional lower intensity Bragg peaks were observable at higher *q* in synchrotron SAXS data (Figure 2A). Notably, in all cases, the higher-order peaks were positioned at *q*/q* = √3 and 2 (where q* is the position of the primary scattering peak), and in some cases, an additional peak at *q*/q* = √7 (see Figure S6 for peak indexing). These signature patterns strongly indicate the presence of a hexagonal cylinder phase. In one case (**4N**_**C**_**12**), an additional peak was observed that did not fit with an expected reflection for the hexagonal phase, and when combined with other peaks, was not consistent with any other kind of unit cell (e.g., cubic). This suggests the coexistence with another LC phase or crystalline phase, which is not possible to determine. The two major exceptions to this nanostructure-activity trend were **4N′12**, which showed a well-defined hexagonal phase within the particles (Figure S7B) but the MIC was 50 μM, and **4N′8**, which showed no evidence of structure but possessed a low MIC at 2 μM.

Inverse hexagonal phases were observed in our earlier study in lipidoids protonated by either sulfuric or hydrochloric acids.^24^ However, the phase was only previously seen for protonated lipidoids with nonlinear tails and branched headgroups. In addition, such protonated lipidoids more readily formed the double gyroid phase, a cubic phase consists of interpenetrating three-dimensional (3D) networks with low interfacial curvature relative to the hexagonal phase. Here, the lipidoids observed in the hexagonal phase had linear tails of intermediate lengths (9–12 atoms long) and linear, branched, or cyclic headgroups. In addition, the phases observed here comprise much larger hexagonal unit cells (*a*_hex_ = 6–6.2 nm) relative to the hexagonal phases in protonated lipidoids (2–3 nm). For protonated lipidoids, we proposed that lipidoids form inverse LC phases from disc-like conformations that stack together such that hydrophobic tails are splayed in all directions away from a central polyamine core, which comprises the hydrophilic cylinder or cubic network. However, conventional lipids and amphiphiles typically pack into cylinders as inverse conical or wedge conformations (Figure 2C).^35^

In our prior study, the bulky branched and/or cyclic tails were proposed to preclude the formation of conical conformations, forcing the molecules to flatten into discs that stacked into either the gyroid or hexagonal phase. However, in another earlier study, inverse cubic micellar phases were observed in lipidoids comprising the **3N**_**B**_ headgroup substituted with 6 linear alkyl epoxide tails. In these highly curved phases, inverted conical conformations are also required. The differences in the chemical structure of lipidoids forming hexagonal phases in LB medium relative to those in acid allude to different molecular conformations and the influence of the surrounding medium. The ca. 2-fold larger dimensions measured here with lipidoids of similar molecular dimensions could be rationalized by swelling of the hydrophilic core by water and the presence of diverse ions and biological molecules from the growth medium. This would not be possible in a disc-like lipidoid conformation, where the headgroup bridges the cylinder and defines its diameter; hydration could only lead to increasing the distance between stacks and reducing intermolecular correlation over long ranges. Thus, for lipidoids with 6 linear tails, a conventional conical shape becomes more energetically favorable than the flat disc conformation. Notably, these dispersions of liquid crystalline particles could be described as hexosomes,^36^ while the particles comprising disordered phases may consist of a dispersed sponge-like structure, as observed in other systems of dispersed amphiphile aggregates.^37^

In the case of the methylated lipidoids which lacked well-defined structures, the molecules are evidently unable to adopt conformations that allow regular packing into LC phases. Here, this phenomenon was observed in two different cases: lipidoids with linear or branched headgroups and 6 bulky nonlinear tails or lipidoids with a cyclic headgroup and 4 short linear tails. In the case of compounds with 6 bulky nonlinear tails, a conical conformation is unfavorable due to steric considerations, as previously rationalized for protonated lipidoids.^24^ Such a lipidoid might instead flatten and adopt a disc-like or chair-like conformation (as present in protonated lipidoid lamellar phases) to maximize spacing between bulky groups. However, due to the presence of numerous components of an aqueous growth medium (including peptides, amino acids, and salts), stacking of the disc or chair-like molecules into well-defined LC morphologies, as observed when protonated in acid, is not possible. Thus, the nonconical shape of these lipidoids may loosely be associated with hydrocarbon-rich and aqueous rich regions comprising no order, leading to the broad peaks observed by SAXS. In the second case, lipidoids with short linear tails would not possess sufficient intermolecular van der Waals’ attractions to allow their aggregation into well-defined LC phases or may instead become fully solubilized in the medium.

In summary, the preferred conformation of lipidoids is evidently a key factor correlating with its antimicrobial activity.

### Bacterial Membrane Permeabilization Correlates to Antimicrobial Activity

The mechanism of action of known cationic and amphiphilic antibacterials (i.e., AMPs and QACs) is usually proposed to be through targeting and disrupting membranes.^30^ To investigate whether cationic lipidoids can disrupt bacterial membranes, we conducted propidium iodide (PI) permeation assays.^38^ PI assays reveal when a membrane becomes compromised by an active compound, as fluorescence is only observed when the dye enters the cytoplasm and interacts with DNA. For *B. subtilis*, a slight increase in fluorescence was observed even in the negative control experiment in the absence of lipidoids, suggesting that the cationic dye itself can slowly access the cytosol under these conditions. Nevertheless, the fluorescence increase caused by lipidoids relative to PI alone provided a metric to quantify membrane permeabilization.

**Figure 3:**
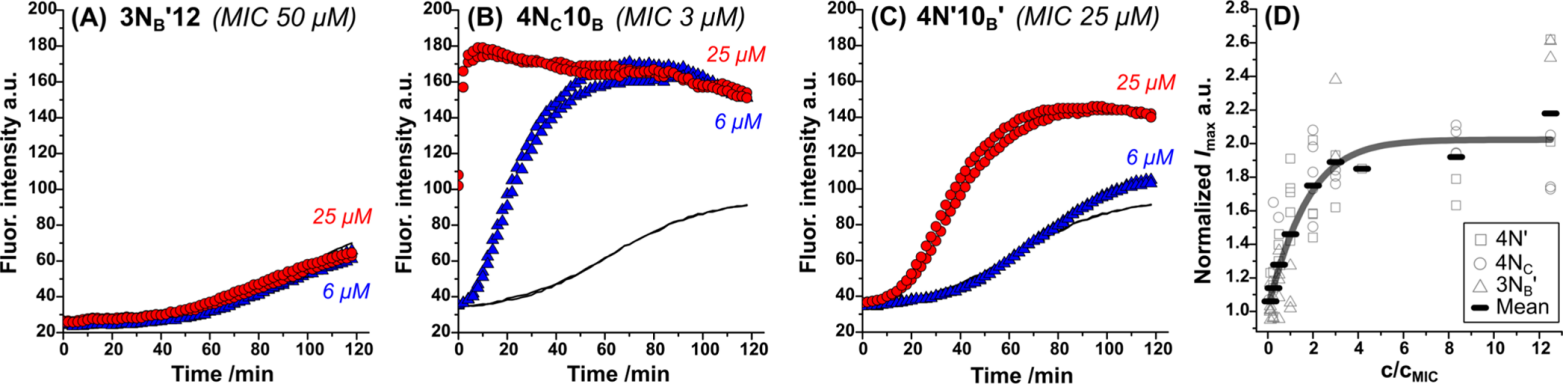
(A–C) Results from representative PI assays performed in duplicate against *B. subtilis* with 25 μM (red circles) and 6 μM (blue triangles) lipidoid and compared to control experiments (black lines). Increasing fluorescence indicates increased cell permeabilization as the dye enters the cell and binds to DNA. Each plot is labeled with the MIC of the lipidoid used. (D) Maximum fluorescence intensity (*I*_max_) plotted against the concentration of lipidoid with respect to the MIC of a given compound (*c*/*c*_MIC_). The overall correlation of *I*_max_ as a function of *c*/*c*_MIC_ strongly indicates that membrane permeabilization is key to antimicrobial activity.

PI assays were performed against 10^7^ CFU/mL *B. subtilis* in the presence of the 27 lipidoids listed in Table 1. Higher concentrations of cells were used for this assay than for MIC assays in order to enhance the intensity of the resulting fluorescence signal. Notably, this would change the lipidoid partitioning equilibrium between the membrane and the medium, but as the results demonstrate, there was still a significant effect on cell membranes. In addition, MBC assays performed at this cell density also demonstrated that lipidoids were still active under these conditions. Measurements showed that for compounds with MIC > 25 μM, fluorescence typically did not increase significantly above the control experiment at either studied concentration (Figure 3A). Meanwhile, for compounds with MIC at 25 μM, an increase in fluorescence was usually observed at 25 μM but not at 6 μM (Figure 3C). Finally, for the most effective compounds with MIC < 6 μM, an increase in fluorescence was seen at both concentrations (Figure 3B). The main exceptions were **4N′12** and **4N**_**C**_**8**, which showed some extent of permeabilization at 25 μM, despite MIC values at 50 or 100 μM, respectively (see Figures S8 and S10). Results also revealed different rates of fluorescence increase, alluding to differences in the kinetics of lipidoid interactions with bacteria. In particular, several compounds demonstrated instantaneous activity, achieving maximum fluorescence within 10 min of adding the dye (e.g., Figure 3B).

Plotting the normalized maximum PI fluorescence intensity (*I*_max_) against the concentration relative to the MIC (*c*/*c*_MIC_) showed a clear increase in *I*_max_ with increasing *c*/*c*_MIC_. This trend appeared universal across all lipidoids studied here, such that all data could be fitted onto a master curve in which *I*_max_ reached a plateau at around *c*/*c*_MIC_ > 3 (Figure 3D). In other words, the extent of permeabilization of bacterial membranes by the lipidoid correlates directly to its antimicrobial activity at that concentration. These results strongly support our hypothesis that cationic lipidoids are membrane-active and their mode of action involves membrane disruption. Moreover, the most rapidly membrane-permeabilizing compounds were those with the tendency to form hexagonal liquid crystal phases within the medium-dispersed particles.

### Membrane Permeation of Mammalian Cells: Erythrocytes

To screen for toxicity against mammalian cells, the activity of cationic lipidoids against human erythrocytes was tested in hemolysis assays.^39^ In these assays, the release of hemoglobin from red blood cells is monitored by ultraviolet–visible (UV–vis) analysis, thus enabling assessment of the extent of membrane disruption caused by active compounds (see the Experimental Section for details). The cell membranes of erythrocytes are asymmetric, with negatively charged lipids mostly localized at the inner leaflet (Figure 5B).^40^ Unlike bacteria, erythrocytes do not contain a cell wall, and their membranes contain cholesterol, a molecule known to modulate the curvature stress within the bilayer.^41^ These featural differences would be expected to result in different interactions of lipidoid molecules with red blood cell membranes and should allow an understanding of the molecular features that lead to selectivity.

**Figure 4:**
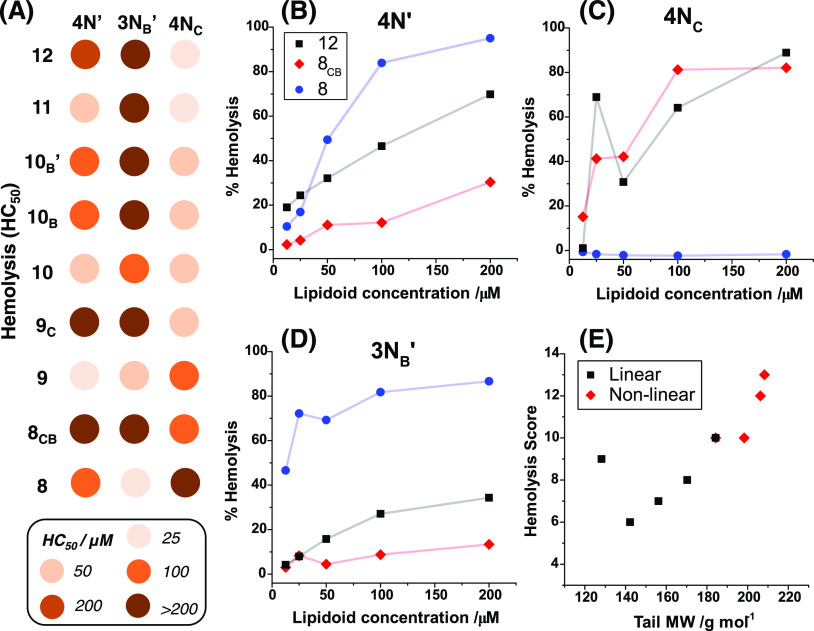
(A) Summary of results from hemolysis assays of 27 lipidoids, with spots colored according to minimum concentrations at which >50% hemolysis had occurred (HC_50_). Examples of hemolytic dose–response curve data for lipidoids comprising (B) **4N′**, (C) **4N**_**C**_, and (D) **3N**_**B**_**′** headgroups with tail groups labeled therein. (E) Plot showing the trend observed between hemolytic score and tail molecular weight.

Hemolytic assays were conducted on the smaller library of 27 lipidoids at various lipidoid concentrations up to 200 μM and compared for HC_50_ values (the lowest concentration at which >50% hemolysis occurred). HC_50_ values are summarized in Figure 4A alongside representative raw dose–response curves (Figure 4B–D). As with MIC assays, the hemolytic activity varied significantly with the lipidoid structure, and the architectures of the head and tail groups were evidently important factors. Lipidoids based on the cyclic **4N**_**C**_ headgroup showed the overall highest toxicity, with HC_50_ values <50 μM for almost every tail, and higher values only observed at shorter tail lengths. Meanwhile, **3N**_**B**_**′**-based lipidoids showed the lowest hemolysis for almost every tail group and a tendency for higher toxicity with shorter tails. For **4N′** lipidoids, lower hemolytic activity was typically observed for compounds with nonlinear tails. Each compound was scored with a number proportional to its HC_50_ concentration, i.e., 0 for 12.5 μM, 1 for 25 μM, 2 for 50 μM, 3 for 100 μM, 4 for 200 μM, and 5 for >200 μM. From this system, total scores for each tail showed a strong negative correlation between hemolytic activity and tail molecular weight, with heavier branched tails demonstrating the lowest hemolytic activity (Figure 4E). This trend indicated that nonlinear tails (**8**_**CB**_, **9**_**C**_, **10**_**B**_, **10**_**B**_**′**) demonstrate significantly lower hemolytic activity than their linear counterparts. Hemolysis was also closely correlated with lipidoid conformation and aggregate structures. In general, lipidoids for which well-defined self-assembled structures in LB were observed were also significantly more toxic than lipidoids that lacked liquid crystalline phases.

Comparing antibacterial and hemolysis data for these 27 lipidoids allowed us to assess structures with the greatest selectivity to bacterial cells over mammalian cells, or therapeutic index (TI), defined here as the ratio of HC_50_ to MIC (Table 2). Although many of the compounds that were active against bacterial cell membranes also readily lysed red blood cells, several compounds emerged that opposed the trend and demonstrated higher selectivity. Despite their significantly different structures, two of the most active lipidoids **4N′8** and **4N**_**C**_**8**_**CB**_ (MIC = 2 μM) recorded the highest therapeutic index of 50.

**Table 2:** Summary of Therapeutic Index (TI) Values for the 27 Selected Lipidoids

TI (HC_50_/MIC)	headgroup		
tail group	**4N′**	**3N_B_′**	**4N_C_**
**12**	4	≥8	8
**11**	8	≥8	8
**10**_**B**_**′**	4	≥8	25
**10**_**B**_	4	≥8	17
**10**	17	8	17
**9**_**C**_	≥32	≥16	25
**9**	13	25	8
**8**_**CB**_	≥16	≥16	50
**8**	50	13	4

**Figure 5:**
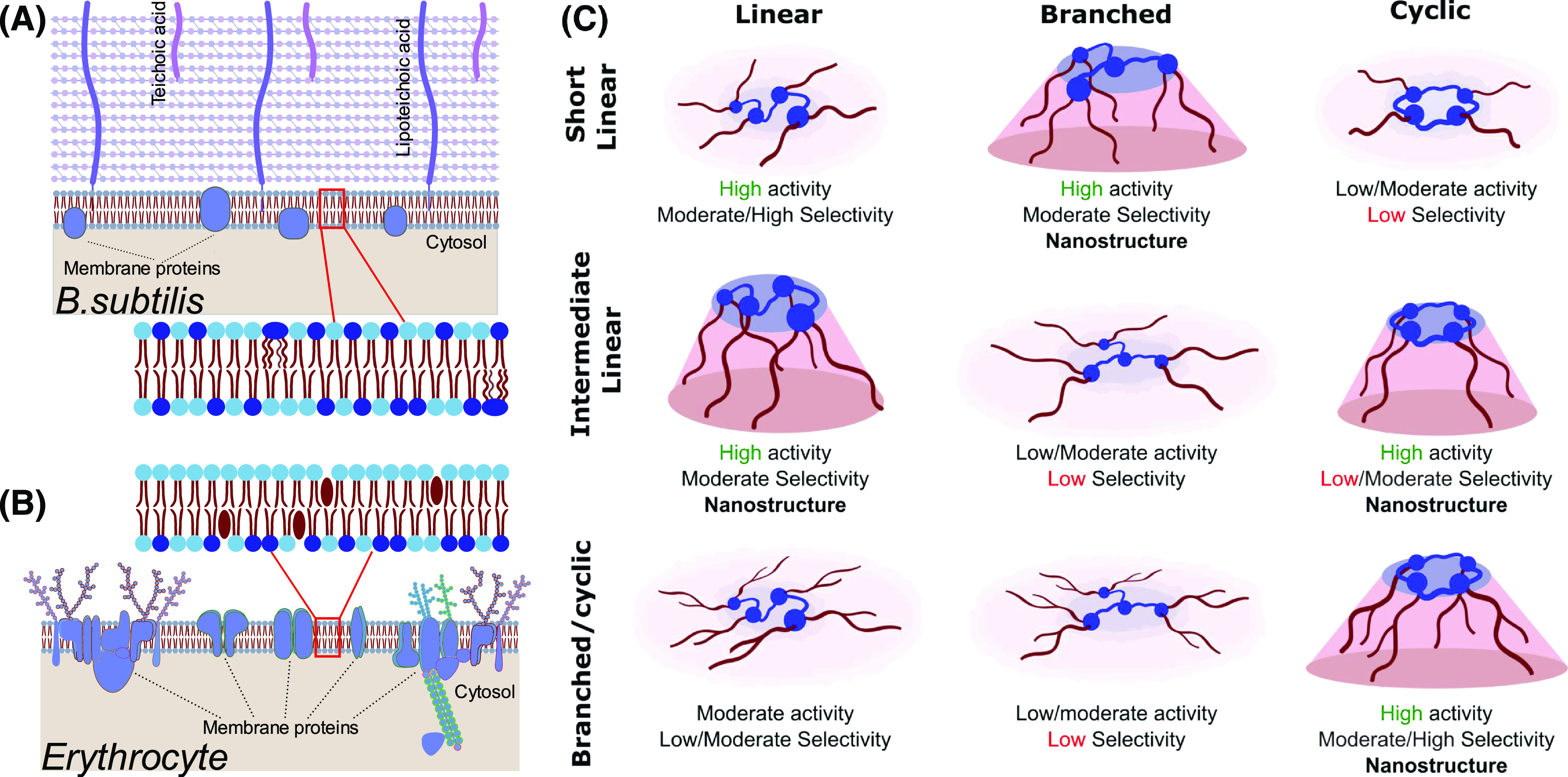
Schematic demonstrating the difference between membrane structures of (A) Gram-positive bacteria *Bacillus Subtilis* and (B) mammalian erythrocytes,^42^ with negatively charged lipids colored with dark blue headgroups. (C) Proposed conformations of structurally different lipidoids, correlated with their antibacterial activities and selectivity over erythrocytes.

The combination of data from MIC assays, nanostructural analysis by SAXS, permeabilization, and hemolytic assays builds a physical picture of the driving forces for membrane-disrupting action of lipidoids. The activity and selectivity of different classes of lipidoids with respect to their observed nanostructure and proposed conformation are outlined in Figure 5C. In general, the charged amine headgroup of cationic lipidoids should interact electrostatically with phospholipids via the phosphoric headgroups, but the differences in arrangement of apolar volume appear to be more crucial in determining membrane disruption. The strong correlation between the lipidoid nanostructure in growth medium and its antimicrobial activity against *B. subtilis* at the lowest concentrations indicates the ability of these lipidoids to induce negative curvature to drive defects or pores within the membrane structure, which allows permeabilization.^43^

Notably, the thick cell wall should be considered as the first barrier protecting bacterial cells. The network of peptidoglycan comprising the cell wall contains pores up to 60 nm in diameter^44^ and has been found to be permeable to macromolecules of up to 50 kDa,^45^ i.e., much smaller than the dimensions of lipidoid aggregates within the medium. However, the cell wall of *B. subtilis* was not previously found to prevent the activity of other hydrophobic compounds with a tendency to form aggregates, including OCT^18^ and hydrophobic AMPs.^12^ We speculate that when lipidoid aggregates collide with bacteria, they will dissociate, assisted by interactions with anionic components in the cell wall (e.g., [lipo]teichoic acid), and diffuse through the network as smaller aggregates or even monomers to reach the cytoplasmic membrane.

Most of the lipidoids unable to form hexagonal phases were poorly membrane disrupting, less antibacterial, and less toxic. It is likely that these lipidoids possess a conformation that is closer to the disc-like or chair-like conformations seen in protonated lipidoids, which might interact less readily with bilayers, and/or their mechanism of disruption may differ (evidently, they were still active at higher concentrations). There were two notable exceptional cases: the lipidoid **4N′8**, which showed no structure by SAXS but was active at 2 μM against *B. subtilis*, and **4N′12**, which showed a hexagonal structure by SAXS but was only antimicrobial at 50 μM. In the former case, the very short tail length may have precluded LC assembly, even if the molecules were able to adopt conical conformations. In the latter case, the longer tail length may have allowed for more favorable insertion into the membrane phospholipids, such that the molecules are easily incorporated without leading to large-scale membrane disruption. It is important to note that conformations in the presence of cell membranes could differ from those observed in aggregates dispersed in growth medium. Noncovalent interactions with phospholipids and other membrane components would likely affect how tails arrange with respect to the headgroups. By whichever means the lipidoid interacts with the membrane, PI permeabilization assays demonstrated that they introduce membrane defects, which could lead to cell death by membrane disruption or by providing access of lipidoids to the cytoplasm, where they can, for example, bind to DNA.

We note that the full library of lipidoids were also tested against Gram-negative *E. coli* bacteria, as outlined in a separate study,^46^ and this species was found to be less susceptible to almost every lipidoid compound relative to *B. subtilis*. This observation further highlights the importance of membrane structure in lipidoid mode of action: Gram-negative bacteria have a second outer membrane comprising structurally distinct lipids that provide an additional barrier against permeation by lipidoids.

Overall, our findings here indicate that membrane-targeting antimicrobial efficacy may be related to the ability of an amphiphile to adopt molecular conformations that disrupt the membrane bilayer, a finding which could be universal amongst structurally related compounds, i.e., QACs. These interactions of lipidoid aggregates with the bacterial membrane resembles how AMP secondary structures are key to their membrane activity, with α-helical compounds particularly prone to disrupt the lipid bilayer.^47,48^ To the best of our knowledge, such a correlation between the aggregate nanostructure or molecular conformations of synthetic compounds and their membrane-targeting antimicrobial activity has not previously been reported. Interestingly, the results presented here resemble the connection between the conformation of the membrane-anchoring region of lipopolysaccharides found in Gram-negative bacteria outer membranes (lipid A) and its endotoxic activity against eukaryotic cells.^49−51^

QACs already find use within disinfectants^52^ and as biofilm-disrupting compounds.^53^ Results here suggest that lipidoids could be applied in these contexts and also within other antimicrobial formulations, e.g., to potentiate hydrophobic drugs that are unable to penetrate into the intracellular space alone. In broader healthcare contexts, the self-assembled morphologies of lipids used for gene delivery are known to affect efficacy, where fusogenic amphiphiles with a propensity for nonlamellar phases are found to enhance membrane-driven endosomal escape.^54,55^ Gene delivery vectors based on cationic lipid-like compounds have already been successfully applied for COVID-19 vaccines, and the technology is anticipated to have broader implications across diverse fields in healthcare.^56^ Therefore, these studies into antimicrobial lipidoids show promise for a range of applications in which membrane permeabilization is key to the mechanism of action.

## Conclusions

Establishing relationships between the molecular structure and membrane activity of synthetic amphiphiles is an important step toward numerous biological applications, in particular understanding antibacterial activity and designing next-generation selective antimicrobials that can overcome the development of resistance. Here, we used cationic lipidoids as a modular platform of materials to explore the structural features that enable high activity against Gram-positive bacteria and selectivity over mammalian cells. Combinatorial screening of 104 lipidoids initially revealed strong correlations between activity and tail hydrophobicity, with tails of intermediate c log *P* values (3–4), displaying the highest efficacy. We uncovered 7 compounds with antimicrobial activity against *B. subtilis* at concentrations as low as 2 μM. These most active lipidoids were observed by SAXS analysis to adopt molecular conformations that enabled their packing into highly curved inverse hexagonal liquid crystal phases under MIC assay conditions. We demonstrated using dye permeabilization assays that the most active lipidoids also induced the highest membrane permeabilization, indicating that membrane disruption induced by lipidoids is key to their activity. Therefore, the ability of lipidoids to form inverted conical conformations that drive inverted phases is evidently a strong driving force for the disruption of lipid packing within the membrane bilayer. In this way, lipidoids could introduce defects or pores into the membrane that would lead to membrane leakage and allow access of lipidoids to the cytosol, both of which could result in cell death. Lipidoids possessing these shapes also tended to demonstrate higher toxicity toward mammalian cells, however, the screening study revealed two lipidoids with hemolytic toxicity at 50x higher concentration than antibacterial activity. The outcomes of this study provide significant inspiration for the design of future antimicrobials or membrane-active compounds in general for next-generation treatments with various target cells.

## Experimental Section

### Experiment

#### Materials

*tert*-Butyl acrylate (**6**_**B**_, 98%, Sigma-Aldrich), *n*-butyl acrylate (**8**, 99%, Sigma-Aldrich), pentyl acrylate (**9**, 95%, abcr), isobornyl acrylate (**8**_**CB**_, 85%, Alfa Aesar), dicyclopentanyl acrylate (**9**_**C**_, 95%, TCI Chemicals), hexyl acrylate (**10**, 98%, Sigma-Aldrich), 3,5,5-trimethylhexyl acrylate (**10**_**B**_, technical grade, Sigma-Aldrich), 2-ethylhexyl acrylate (**10**_**B**_**′**, 98%, Sigma-Aldrich), heptyl acrylate (**11**, 96%, abcr), octyl acrylate (**12**, 98%, TCI Chemicals), isodecyl acrylate (**13**_**B**_, TCI) decyl acrylate (**14**, 95%, abcr), dodecyl acrylate (**16**, 90%, Sigma-Aldrich), 1,8-diaminooctane (**2N**, 98%, Sigma-Aldrich), tetraethylene triamine (**3N**, 99%, Sigma-Aldrich), spermidine (**3N′**, 99%, Sigma-Aldrich), hexaethylene tetraamine (**4N**, 97%, Sigma-Aldrich), spermine (**4N′**, 99%, Sigma-Aldrich), tris(2-aminoethyl)amine (**3N**_**B**_, TREN, 96%, Sigma-Aldrich), tris(2-aminopropyl)amine (**3N**_**B**_**′**, 97%, TCI Chemicals), 1,4,8,11-tetraazacyclotetradecane (**4N**_**C**_, 98%, Sigma-Aldrich), ethanol (99.8%, Carl Roth), and Triton-X-100 (1% in water, Carl Roth) were used as received. LB medium and LB agar powders were purchased from Carl Roth and diluted with MilliQ water obtained from an ELGA Purelab flex system. PBS (e.g., 50 mL in milliQ water) was prepared from 0.38 g of NaCl (130 mM), 0.117 g of Na_2_HPO_4_ (water-free), and 0.025 g of NaH_2_PO_4_ monohydrate and adjusted to pH 7.4 or 7 for PI and hemolysis assays, respectively. OCT was supplied by Schülke & Mayr (Vienna, Austria).

#### Lipidoid Synthesis

Reactions were conducted in ethanol at 3.0 M with respect to the total number of moles of amine and acrylate. The acrylate was added in a 1.1 molar excess relative to the total number of reactive amine sites (N). For example, **3N**_**B**_ (TREN) contains 3 primary amines, which can each react with two acrylates (*N* = 6); therefore, the molar ratio of acrylate to TREN was 6.6:1.

##### Representative Synthesis of Lipidoid **3N10**_**B**_

Diethylenetriamine (**3N**, 100 μL, 0.92 mmol) and 3,5,5-trimethylhexyl acrylate (**10**_**B**_, 1.148 mL, 5.06 mmol) were added to a 4 mL glass vial before adding ethanol (1.995 mL) and homogenizing all components using a vortex mixer. The vial was sealed and heated to 37 °C for 96 h while shaking at 200 rpm in a Thermo Fisher MaxQ4450. Following this, most of the solvent was removed on a Techne Driblock DB200/3 sample concentrator (Cole-Parmer, Vernon Hills, IL) under a flow of nitrogen at 40 °C to leave a clear oil. The remaining solvent and unreacted acrylate were removed under vacuum at 65 °C overnight. However, to facilitate a rapid screening approach, we conducted no further purification steps beyond vacuum treatment.

#### Analysis of Purity

^1^H NMR spectroscopy was performed on a Bruker Avance III 300 MHz spectrometer with a BBO probe head. Spectra were recorded as an average of 16 scans and analyzed using Bruker TopSpin 4.1.4. Chemical shifts were referenced to the CDCl_3_ peak at 7.26 ppm. The chemical shifts of peaks observed for most compounds are listed in a prior study,^24^ and the details of new compounds studied here are provided in Table S1. Example spectra are also shown in Figure S1.

The main impurities in the final product were unreacted monomer and lipidoid species with fewer tails substituted. Unreacted monomer could be removed under vacuum, as confirmed by undetectable levels of acrylate protons in ^1^H NMR spectra. By comparing the integral of protons adjacent to nitrogen atoms (either 1 or 2 carbons away, *I*_N_, in the region 2–4 ppm) to the integration arising from the O–CH_2_ or O–CH protons (*I*_A_, in the region 4–5 ppm) from the acrylate tails, the degree of substitution could be estimated, i.e., full substitution (*N*), missing one tail (*N* – 1), or missing two tails (*N* – 2), etc. The theoretical *I*_N_:*I*_A_ value was calculated for every combination of amine and acrylate with every degree of substitution, against which the experimentally derived values could be compared. In the vast majority of cases, the values lay closer to the value for *N* than *N* – 1, indicating that the fully substituted lipidoids were the major product.

#### Bacterial Culture Preparation

WT *B. Subtilis* 168 *trpC*2^18^ was grown to mid log phase and prepared as a frozen stock in glycerol/water 9/1. An aliquot of the frozen stock was streaked out onto an Agar plate prepared with LB medium and grown overnight at 37 °C. A single colony from this plate was then transferred to 3 mL of LB medium and grown for 16–18 h to prepare the overnight culture (ONC). Cell density of the ONC was measured by optical density at 420–580 nm (OD_420–580_), using an ONDA V-10 plus spectrophotometer. An aliquot of the ONC was diluted to an OD value of 0.05 and grown until the mid log phase (3.5–4 h at 37 °C) to produce the main culture (MC). The OD_420–580_ of the MC was measured and 1 mL was pelleted by centrifugation at 4500 rpm for 5 min before washing with PBS buffer twice. Following redispersion in medium or PBS, the main culture was diluted to the desired cell density (calculated based on an OD_420–580_ = 1 equiv to 8.8 × 10^7^ cells/mL for *B. subtilis*). To each well of the 100-well honeycomb plate, 90 μL of this main culture was added. For MIC assays, 1 × 10^6^ cells/mL in LB were used, while for PI permeation assays, a density of 1 × 10^7^ cells/mL in PBS was adopted.

#### Lipidoid Preparation for MIC Assays

Neat lipidoid (ca. 40 mg) was weighed into a tared 2 mL vial and dried under vacuum at 65 °C overnight to remove any residual acrylate, before being dissolved in 0.4 mL of ethanol. To this solution, a 2.2-fold excess of methyl iodide (with respect to total moles of amines) was added, and the solution was allowed to react overnight at 22 °C to enable complete methylation. 40 μL of the ethanolic solution was then added to 0.5 mL of DMSO in a 1.5 mL Eppendorf tube. Ethanol and unreacted methyl iodide were removed under a flow of nitrogen at 40 °C for 1 h using a Techne Driblock DB200/3. The concentration of lipidoid in DMSO was calculated based on the mass remaining after vacuum treatment and the remaining volume of DMSO measured by micropipette (which was typically 0.45–0.47 mL following the evaporation step). The required volume of DMSO solution was added to MHB or LB medium to prepare a 1 mL solution at 1.0 mM. Aliquots of this solution were then diluted with LB medium to 10× the desired value for the MIC assay. A 10 μL aliquot of each concentration of lipidoid dispersion was then added to a well in a multiwell plate and diluted with 90 μL of bacterial stock to afford a final lipidoid concentration of either 100, 50, 25, 12.5, 6, 3, 2, or 1 μM. Each concentration was studied in triplicate, and in cases where results differed between the 3 wells, the majority result was selected. In each individual assay, a positive control (*B subtilis* in LB medium at 1 × 10^6^ cells/mL) and negative control (*B. subtilis* with 0.001% w/v OCT) were also run in triplicate. The MIC assays of 27 lipidoids at lower concentrations were also repeated with two different batches of lipidoid.

#### Bacterial Growth Calibration Curve

Bacteria were grown overnight in LB medium as described above, and after preparing the main culture, it was diluted to 1 × 10^7^, 1 × 10^6^, 1 × 10^5^, 1 × 10^4^, and 1 × 10^3^ cells/mL. 100 μL of each cell density was added to three separate wells on a 100-well honeycomb plate. The cultures were then grown at 37 °C in a Bioscreen C MBR (Bioscreen, Turku, Finland) and growth was followed by measuring OD_420–580_ over a period of 24 h. To determine the onset of the log phase for different cell densities, the first linear region of OD increase was fit by linear regression (Figure S3B). The time point at which the fitted line intersected with the baseline (determined by the OD value before the log phase began) was then defined as *t*_onset_. A plot of *t*_onset_ vs cell density could be fit with a straight line, which allowed the correlation of onset time for growth with cell density. Thus, for each MIC assay, the delay of onset time in the presence of lipidoid relative to the positive control experiment (*B. subtilis* only) could be used to estimate the percentage of bacterial cells inhibited by lipidoids. For our assays, MIC was defined as the concentration of lipidoid at which 99.9% of bacteria was inhibited, which correlated with a delay of >150 min relative to *B. Subtilis* alone.

#### MBC “Copacabana” Assay

After establishing MIC values, a number of lipidoids were also investigated for MBC (minimal bactericidal concentration). Bacterial suspensions were prepared as described above in LB medium at a density of 1 × 10^7^ CFU/mL and incubated with lipidoids at 6.25, 12.5, and 25 μM for 1 h at 37°C in a honeycomb plate. For control experiments, serial dilutions of *B. subtilis* were prepared (10^2^ and 10^3^ CFU/mL) and also incubated at 37 °C for 1 h. Following incubation, a 100 μL aliquot of each well was dispensed onto the middle of a solid LB agar plate, and autoclaved molecular glass beads with a diameter of 5 mm were added. The lid of the Petri dish was closed, and the beads were rolled over the entire surface of the plate under constant circular shaking for 60 s in order to separate and evenly distributed the colonies on the agar plate. The glass beads were then removed before the agar plates were incubated for 24 h at 37 °C to allow living cells to grow. After 24 h, cells were counted and compared to the control experiment. The lipidoid concentration at which the number of viable CFU was reduced by >99.9% within the culture following 1 h incubation with lipidoids and 24 h growing on agar plates was used to evaluate bactericidal activity. In all cases, these MBC values matched exactly with MIC values, despite the higher concentration of cells used. This provided evidence for the ability of lipidoids to kill bacteria within 1 h of exposure.

#### Hemolysis Assays

A mixture of male and female human erythrocytes was obtained from healthy donors at the Universitätsklinik für Blutgruppenserologie und Transfusionsmedizin (MedUni Graz) and supplied as 1 × 10^6^ cells/μL. An aliquot of these erythrocytes was diluted to 5% v/v using PBS. Methylated lipidoids were prepared as suspensions at different concentrations in PBS using the same protocol as for dispersing in LB for MIC assays. In an Eppendorf tube, 75 μL of each lipidoid in medium was added to 75 μL of the 5% erythrocyte solutions such that the final concentrations of lipidoids were 12.5, 25, 50, 100, or 200 μM in PBS, and the final suspension of erythrocytes was at 2.5% (cell density of 2.5 × 10^7^ cell/mL). These mixtures were incubated at 37 °C for 1 h, before being centrifuged at 2,800 rpm for 5 min. The supernatant was transferred to a 96-well honeycomb plate and the absorbance at 450 nm was measured using a Bioscreen C MBR. Hemolysis % was calculated using 1% Triton-X as the positive control (i.e., 100% hemolysis) and using PBS as the negative control.
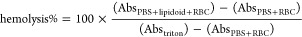
HC_50_ values were measured as the lowest concentration at which more than 50% hemolysis occurred. Lipidoids were measured in two separate hemolytic experiments, which showed reproducible HC_50_ values in every case.

#### Propidium Iodide Permeation Assay

Bacterial cells were grown as described above, and after washing the MC, they were diluted to a concentration of 1 × 10^7^ cells/mL using PBS instead of growth medium. These cell suspensions were then mixed with an aqueous solution of PI to afford a final dye concentration of 2.5 μg/mL. Lipidoid dispersions were prepared as described above in PBS, and 10 μL was added (in duplicate) to a black Flat-Bottom Nunc MicroWell 96-well plate (Thermo Scientific). To each well, 90 μl of bacteria/dye solution was added, such that the final lipidoid concentrations were 25 and 6.25 μM. As a negative control, 10 μL of PBS was mixed with 90 μL of bacteria/dye solution.

#### Small-Angle X-ray Scattering (SAXS)

Lab-source SAXS analysis was conducted on an Anton Paar SAXSpoint equipped with an Excillium liquid Gallium source and a DECTRIS EIGER2 R 1M detector. Dispersed methylated lipidoid samples were loaded into a capillary μ-cell and measured at 37 °C at a sample-to-detector distance of 300 mm, with 6 × 600 s exposures. Background subtraction on lab source data was difficult to achieve due to the relatively low signal-to-noise ratio. Synchrotron SAXS analysis was conducted at the station BM29 at ESRF, Grenoble equipped with a Pilatus3 2M detector at a sample-to-detector distance of 2867 mm. Dispersions of methylated lipidoids in LB medium were prepared and loaded into the capillary at 37.0 °C using an autosampler. 10 × 1 s exposures were collected as the sample moved through the capillary to minimize beam damage. Backgrounds of PBS/DMSO with the same volume ratio used in the final medium were subtracted from the synchrotron SAXS data of lipidoid dispersions.

#### Dynamic Light Scattering

Dispersions of methylated lipidoid in LB were prepared at 2 μM. 1 mL of dispersion was transferred into a polystyrene cuvette and measured on a Malvern Zetasizer Nano ZSP equipped with a 10 mW 632.8 nm laser. Measurements were made at a backscattering angle of 173°, with a lipidoid refractive index set to 1.518 (based on OCT), and the solvent refractive index set to 1.332 and viscosity to 0.7138 cP.

#### Antimicrobial Performance Scoring Criteria

The results of the initial MIC screen were used to generate a score for each combination of head and tail groups. Scores were differentiated based on whether the lipidoid entirely prevented bacterial growth during the experimental timeframe, or whether it delayed growth to an extent that signified 99.9% growth inhibition. Table S1 describes the process used to select scores, and assigned scores are outlined in Table S2. These scores were then plotted against various descriptors as listed in Tables S3 and S4.

## Supporting Information Available

Raw ^1^H NMR data; ^1^H NMR shifts for new lipidoids; scores awarded to each compound in the initial screening assay; quantifying parameters for each head and tail group; DLS data; representative growth curves; additional SAXS data; peak fitting for hexagonal phases; and raw PI assay data (PDF)
