# Supplementary material for: Combinatorial Screening of Cationic Lipidoids Reveals How Molecular Conformation Affects Membrane-Targeting Antimicrobial Activity
Source: ACS Appl Mater Interfaces. 2023 Aug 21;15(34):40178–90. doi: 10.1021/acsami.3c05481 (PMC10472336; doi:10.1021/acsami.3c05481)
Supplement: Supplementary file 1 — am3c05481_si_001.pdf [file am3c05481_si_001.pdf]

Supporting Information

Combinatorial Screening of Cationic Lipidoids Reveals How Molecular Conformation Affects Membrane-Targeting Antimicrobial Activity

James Jennings, Dunja Ašćerić, Enrico Federico Semeraro, Karl Lohner, Nermina Malanovic, Georg Pabst*

Institute of Molecular Biosciences, University of Graz, NAWI Graz, 8010 Graz, Austria
Field of Excellence BioHealth, University of Graz, 8010 Graz, Austria
*E-mail: james.jennings@uni-graz.at

Table S1: List of ^{1}H NMR chemical shifts for new lipidoid structures and measured I_N/I_A values.

Lipidoid structure	Headgroup protons (N-C**H**$_2$, N-CH$_2$-C**H**$_2$…) /ppm	Tail esters (O-C**H**$_2$ or O-C**H**) /ppm	Other tail protons - C**H**$_2$-, -C**H**-) /ppm	Methyl (-C**H**$_3$) /ppm	I_N/I_A calcd	I_N/I_A *(N)*	I_N/I_A *(N-1)*
**2N6$_B$**	2.73, 2.36	X	X	1.44	2.65	2.50	2.67
**2N8**	2.78, 2.44	4.07	1.61, 1.39	0.93	2.49	2.50	2.67
**2N8$_{CB}$**	2.77, 2.40	4.66	1.03-1.89	0.83, 0.98	2.56	2.50	2.67
**2N9**	2.79, 2.44	4.06	1.63, 1.33	0.91	2.44	2.50	2.67
**2N9$_C$**	2.75, 2.39	4.55	0.79 - 2.12	X	2.63	2.50	2.67
**2N10**	2.77, 2.42	4.05	1.60, 1.31	0.89	2.52	2.50	2.67
**2N10$_B$'**	2.77, 2.43	3.98	1.56, 1.35, 1.27	0.89	2.52	2.50	2.67
**2N10$_B$**	2.77, 2.42	4.08	1.01-1.73	0.89	2.51	2.50	2.67
**2N11**	2.78, 2.45	4.05	1.62, 1.29	0.89	2.49	2.50	2.67
**2N12**	2.77, 2.43	4.05	1.61, 1.28	0.88	2.52	2.50	2.67
**2N13$_B$**	2.76, 2.42	4.05	1.61, 1.26, 1.13	0.86	2.44	2.50	2.67
**2N14**	2.76, 2.42	4.05	1.62, 1.26	0.88	2.51	2.50	2.67
**2N16**	2.77, 2.43	4.05	1.61, 1.26	0.88	2.47	2.50	2.67
**3N13$_B$**	2.77, 2.50, 2.43	4.09	1.6, 1.27, 1.12	0.85	2.73	2.80	3.00
**3N'13$_B$**	3.28, 2.98, 2.76, 2.44	4.05	1.61, 1.27, 1.12	0.86	2.79	2.80	3.00
**4N13$_B$**	3.11, 3.03, 2.78, 2.51, 2.43	4.07	1.60, 1.28, 1.13	0.86	3.27	3.00	3.20
**4N'13$_B$**	2.98, 2.77, 2.42	4.05	1.58, 1.26, 1.13	0.86	3.06	3.00	3.20
**2N$_O$13$_B$**	3.57, 2.84, 2.69, 2.47	4.05	1.61, 1.28, 1.14	0.86	3.40	3.50	4.00
**3N$_B$13$_B$**	3.20, 3.02, 2.78, 2.51, 2.43	4.06	1.61, 1.28, 1.13	0.86	3.19	3.00	3.20
**3N$_B$'13$_B$**	3.04, 2.77, 2.55, 2.43, 2.03	4.05	1.58, 1.28, 1.13	0.86	2.97	3.00	3.20
**4N$_C$13$_B$**	2.76, 2.52, 2.43, 1.97	4.06	1.60, 1.27, 1.13	0.86	4.33	4.00	4.67

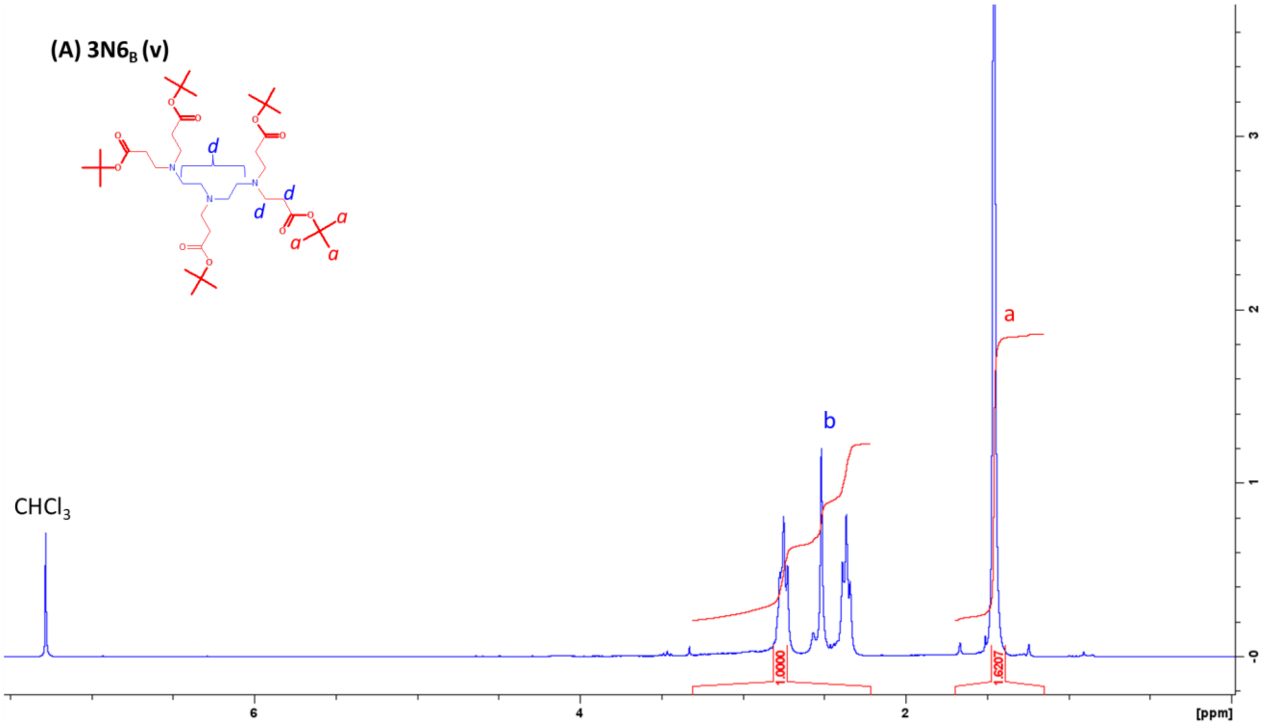
(A) 3N6$_B$ (v)
CHCl$_3$
b
a
1.0000
1.6207
[ppm]

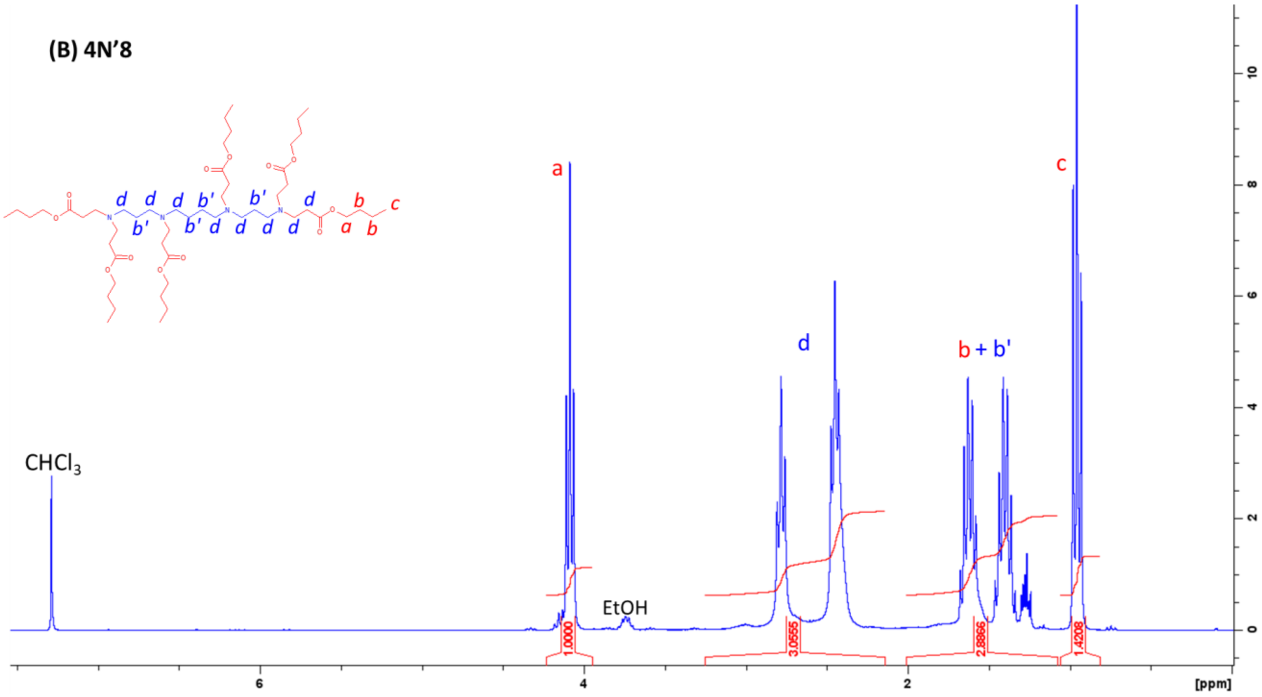
(B) 4N'8
CHCl$_3$
a
EtOH
d
b + b'
c
1.0000
3.0555
2.8866
1.4208
[ppm]

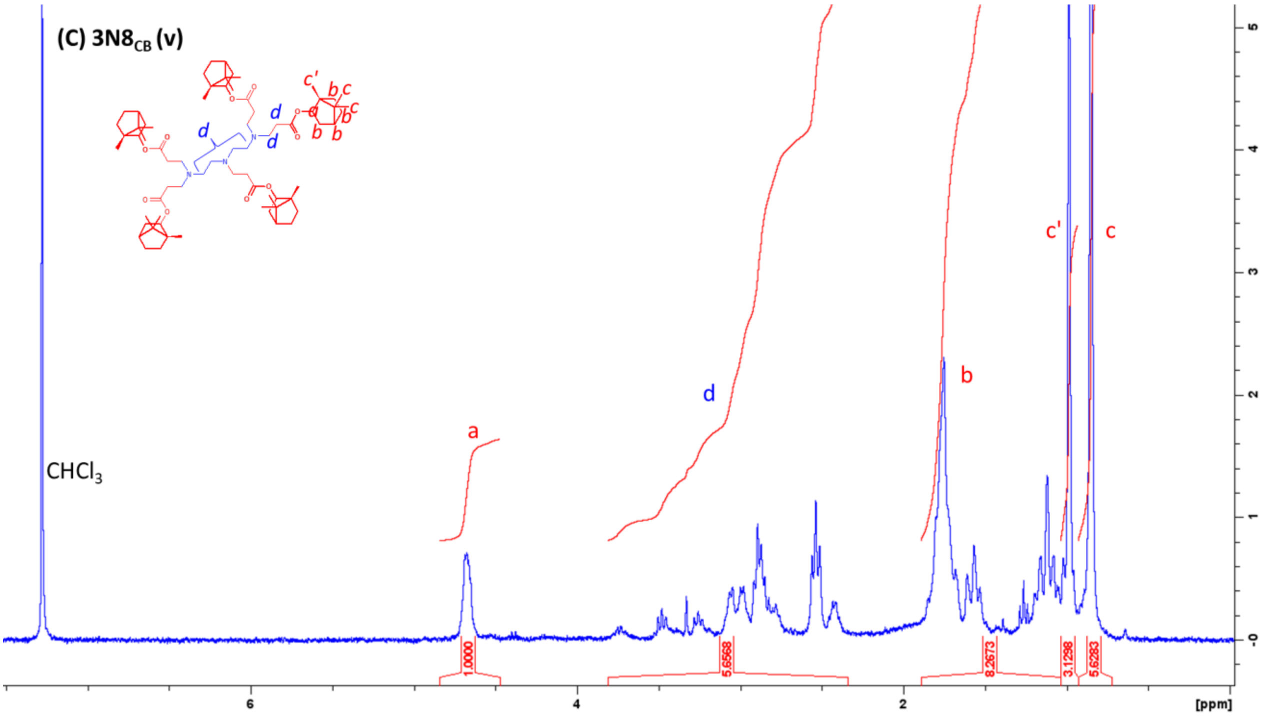

(C) 3N8CB (v)
CHCl3
a
d
b
c'
c
1.0000
5.6568
8.2673
3.1298
5.6283
[ppm]

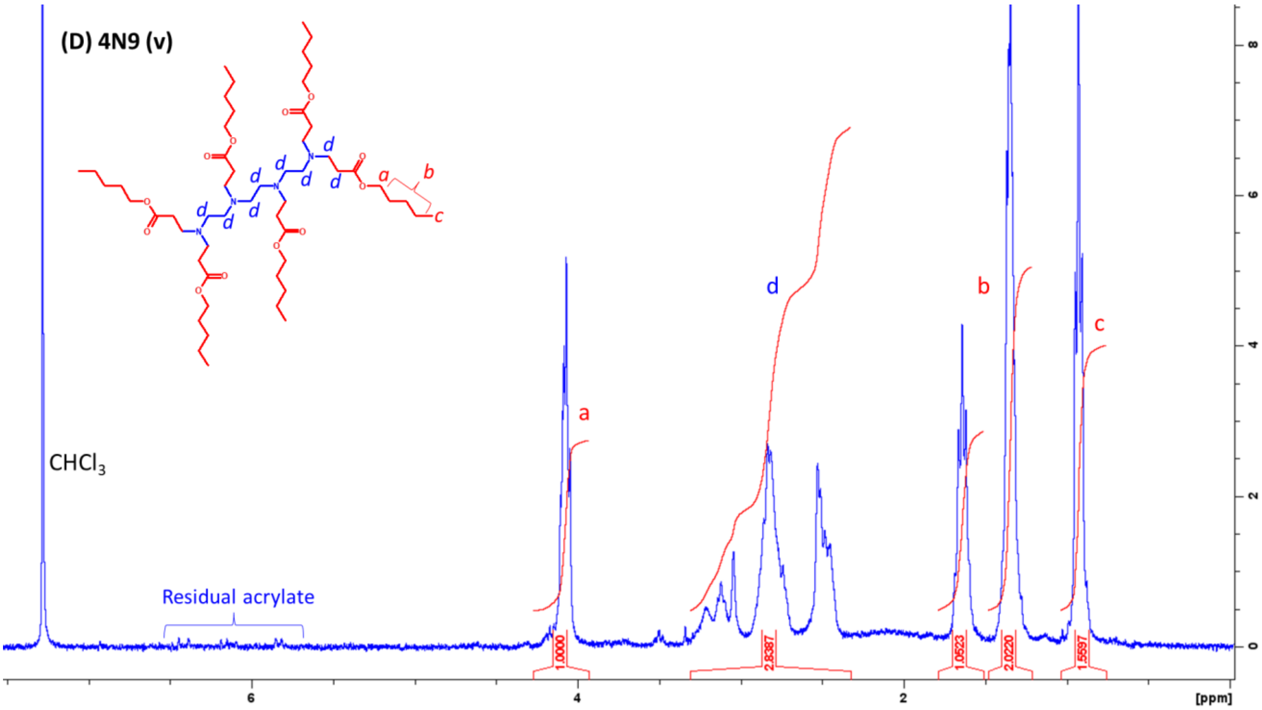

(D) 4N9 (v)
CHCl3
Residual acrylate
a
d
b
c
1.0000
2.8387
1.0523
2.0220
1.5597
[ppm]

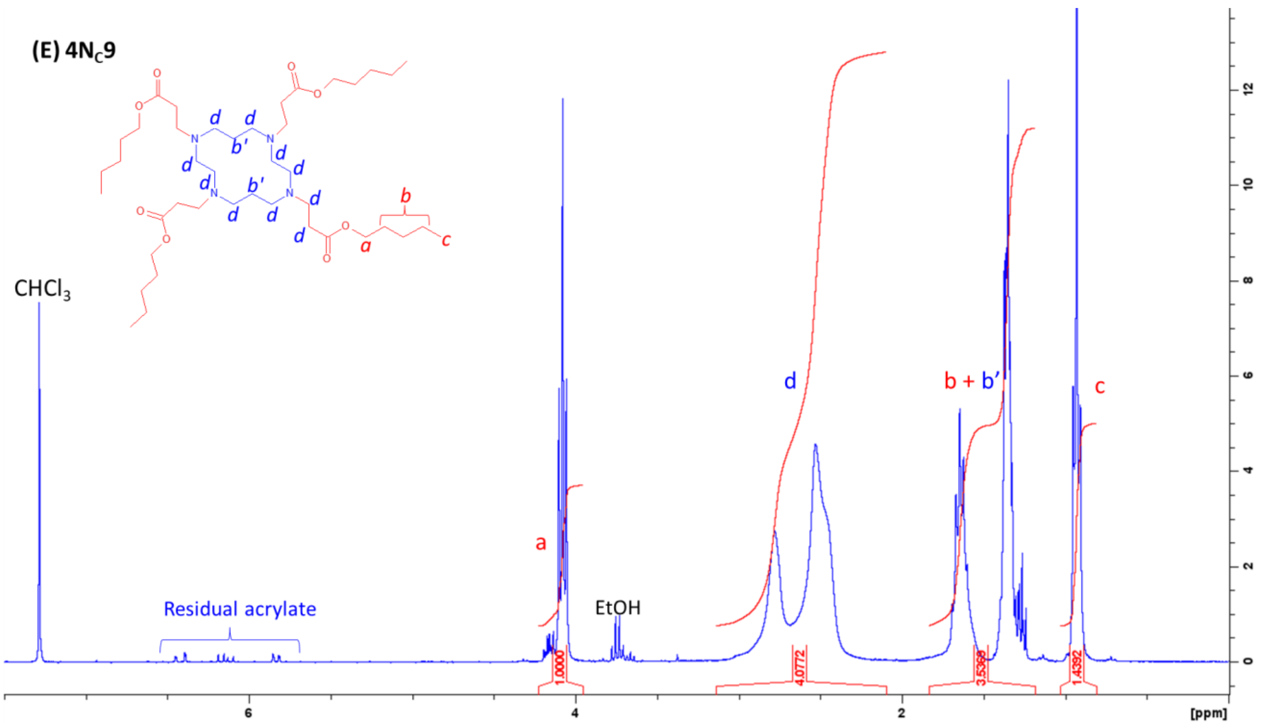
(E) 4N$_C$9
CHCl$_3$
Residual acrylate
a
EtOH
d
b + b'
c
1.0000
4.0772
3.5369
1.4392
[ppm]

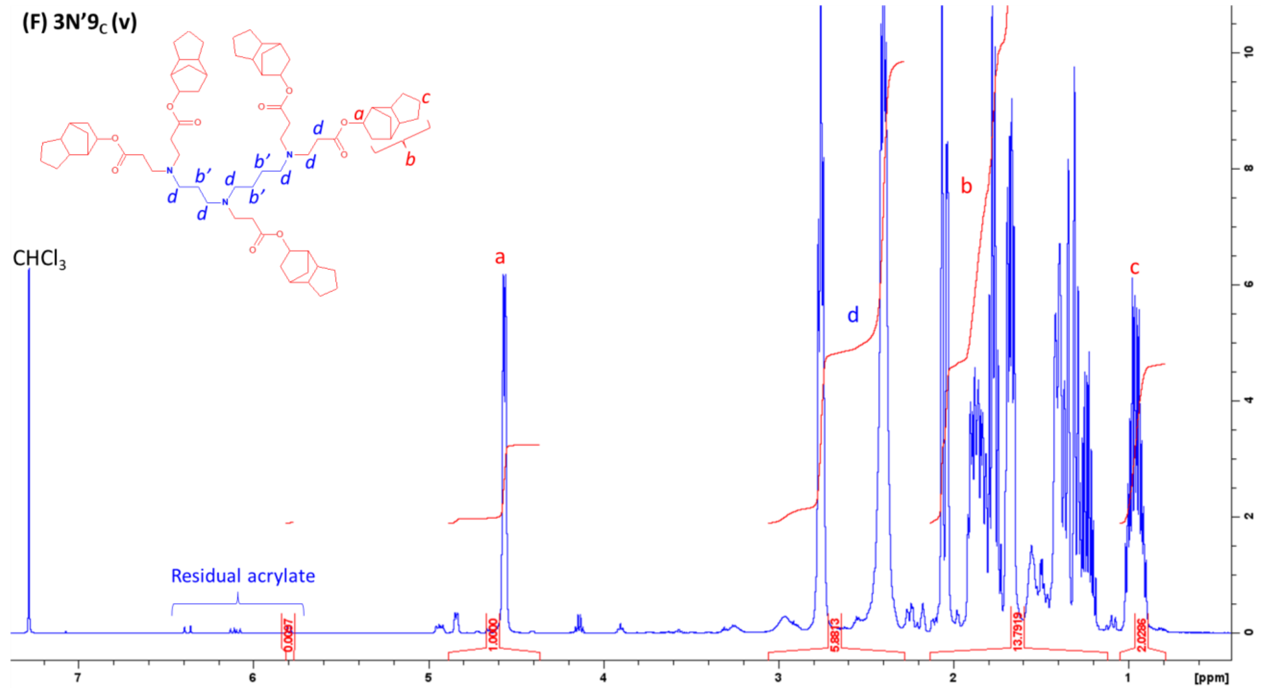
(F) 3N'9$_C$ (v)
CHCl$_3$
Residual acrylate
a
d
b
c
0.0097
1.0000
5.8813
13.7919
2.0286
[ppm]

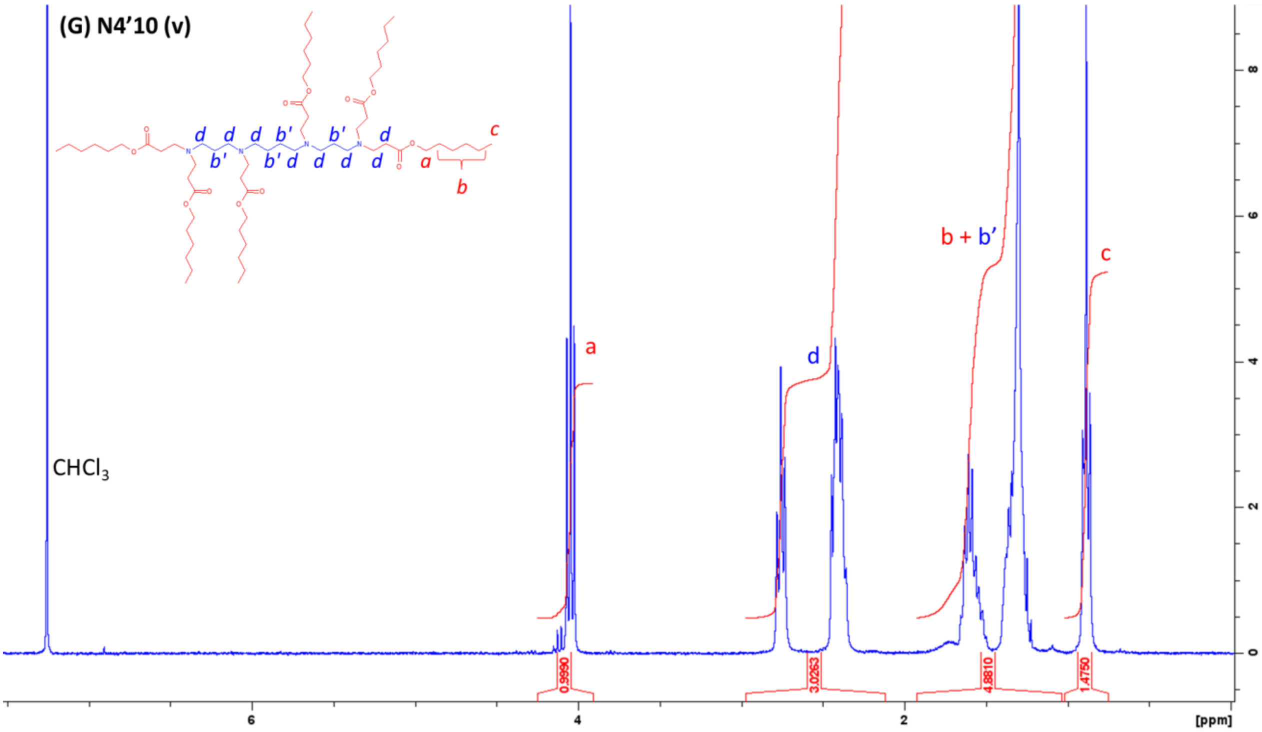

(G) N4'10 (v)
CHCl3
a
d
b + b'
c
0.9990
3.0263
4.8810
1.4750
6
4
2
[ppm]

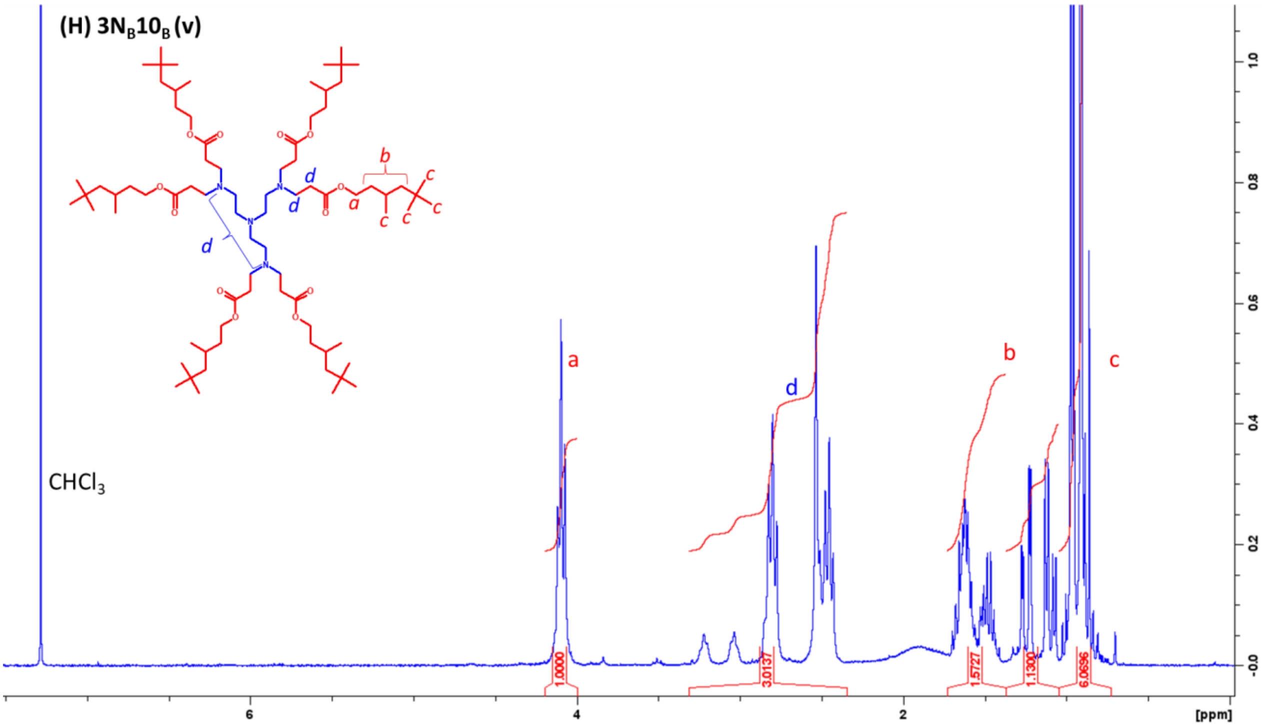

(H) 3NB10B (v)
CHCl3
a
d
b
c
1.0000
3.0137
1.5727
1.1300
6.0696
6
4
2
[ppm]

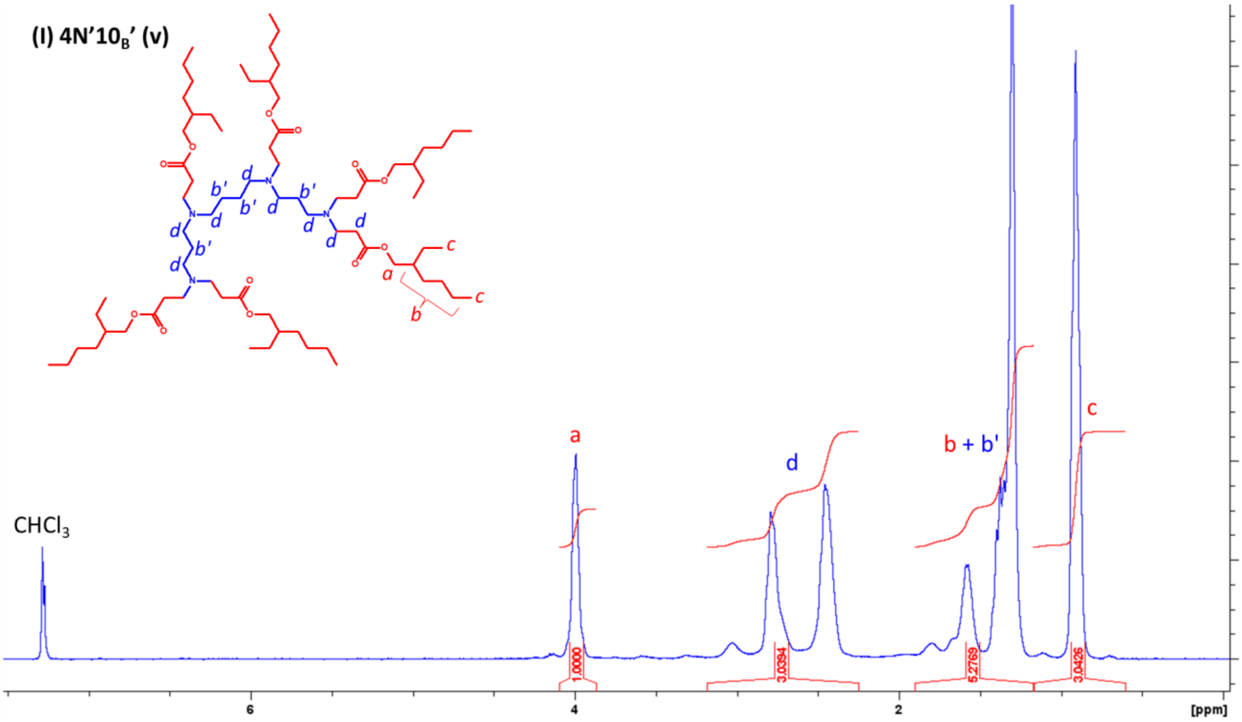
(I) 4N'10B' (v)
CHCl3
a
d
b + b'
c
1.0000
3.0394
5.2769
3.0426
[ppm]

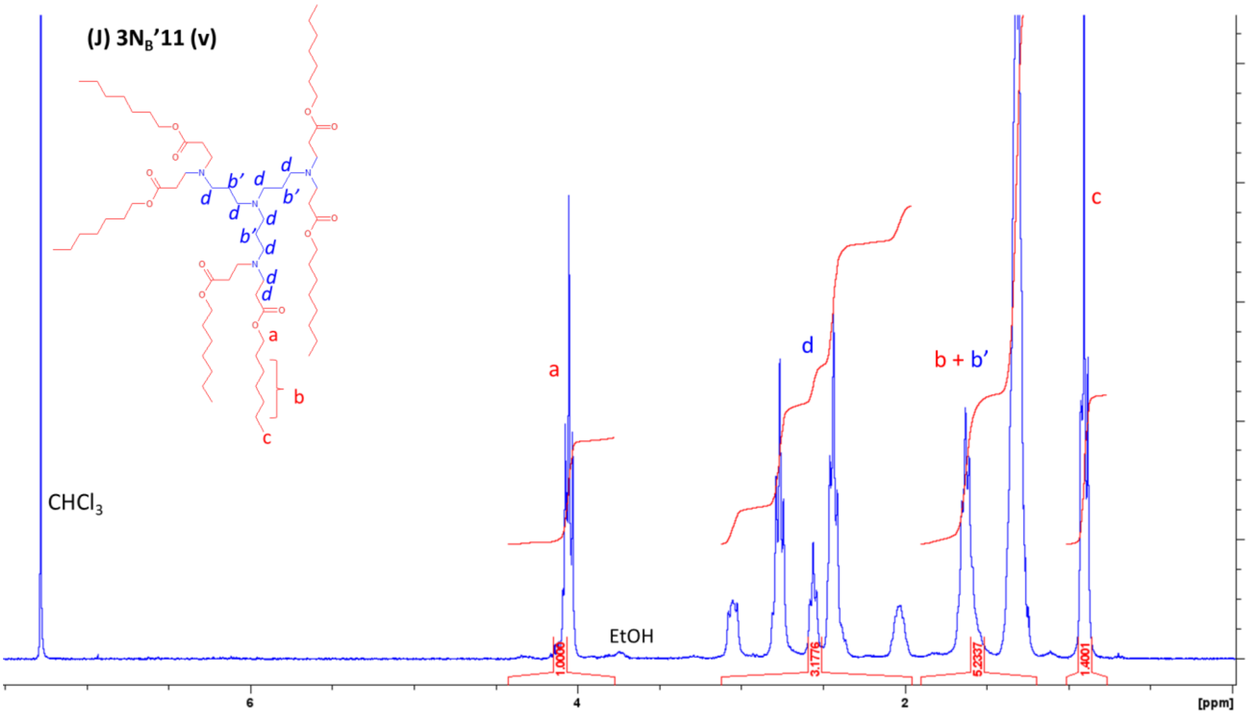
(J) 3NB'11 (v)
CHCl3
a
EtOH
d
b + b'
c
1.0000
3.1776
5.2337
1.4001
[ppm]

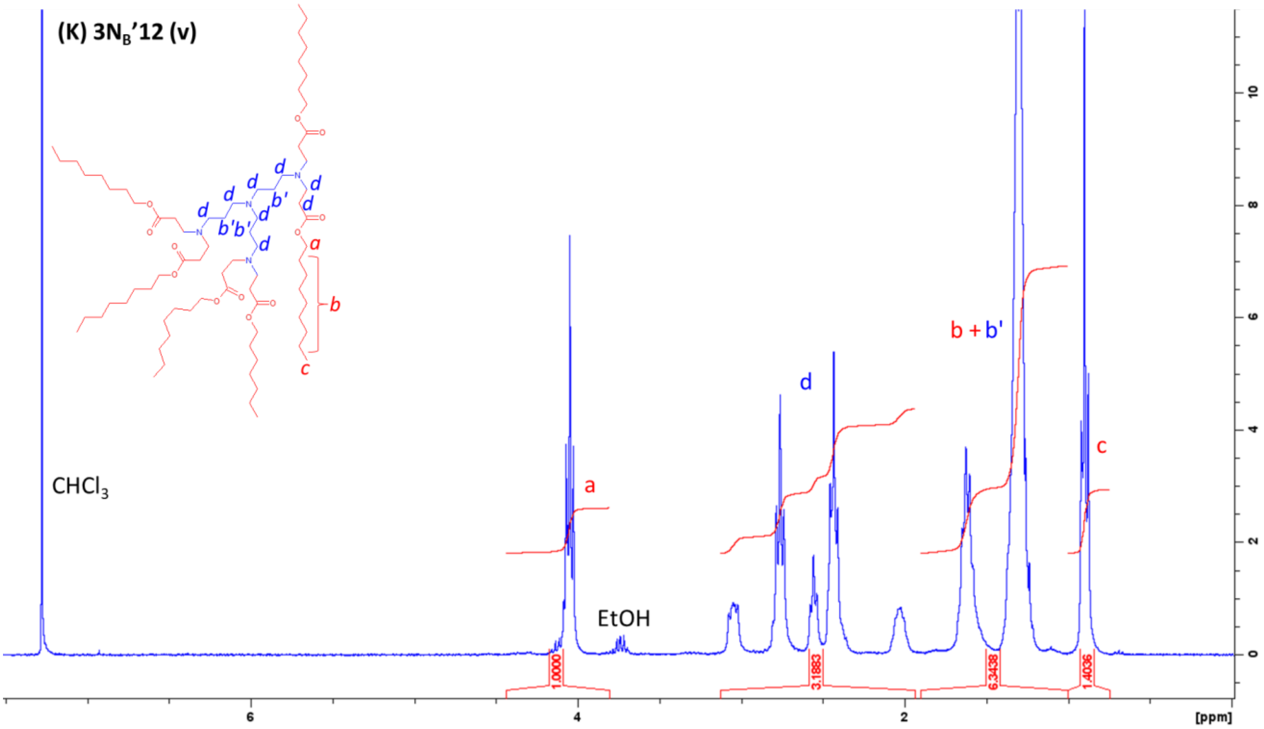
(K) 3N$_B$'12 (v)
CHCl$_3$
EtOH
a
d
b + b'
c
1.0000
3.1883
6.3438
1.4036
6
4
2
[ppm]

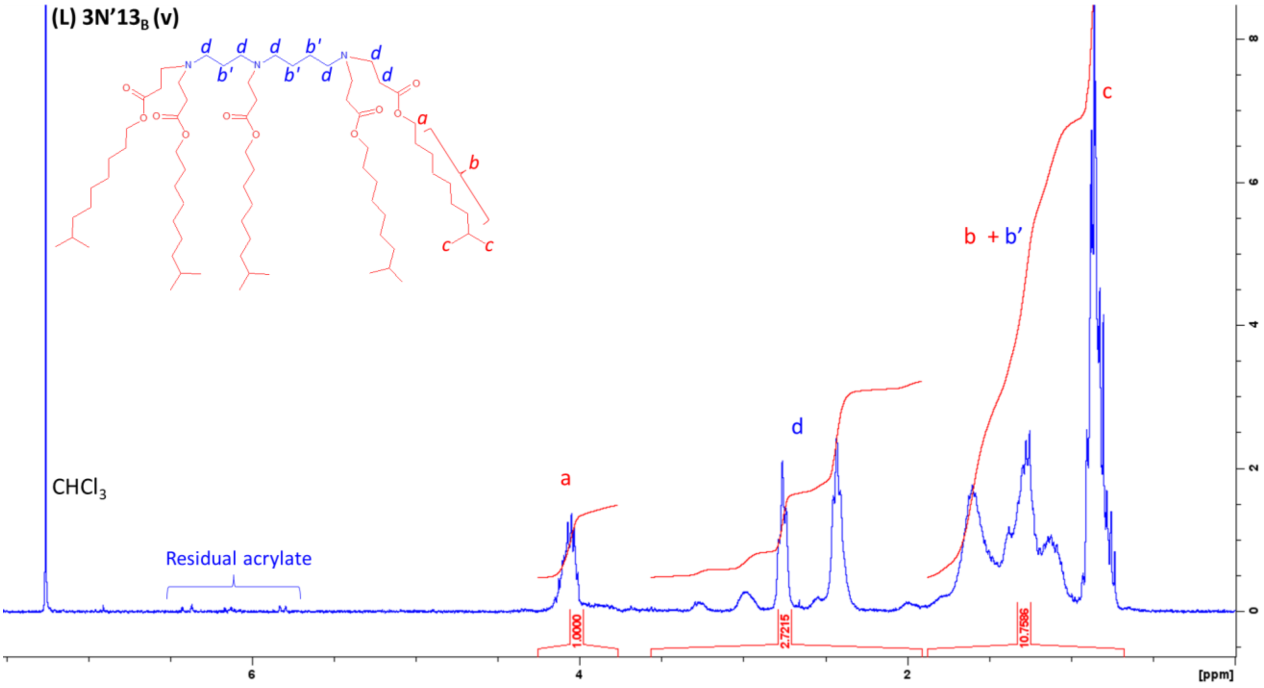
(L) 3N'13$_B$ (v)
CHCl$_3$
Residual acrylate
a
d
b + b'
c
1.0000
2.7215
10.7586
6
4
2
[ppm]

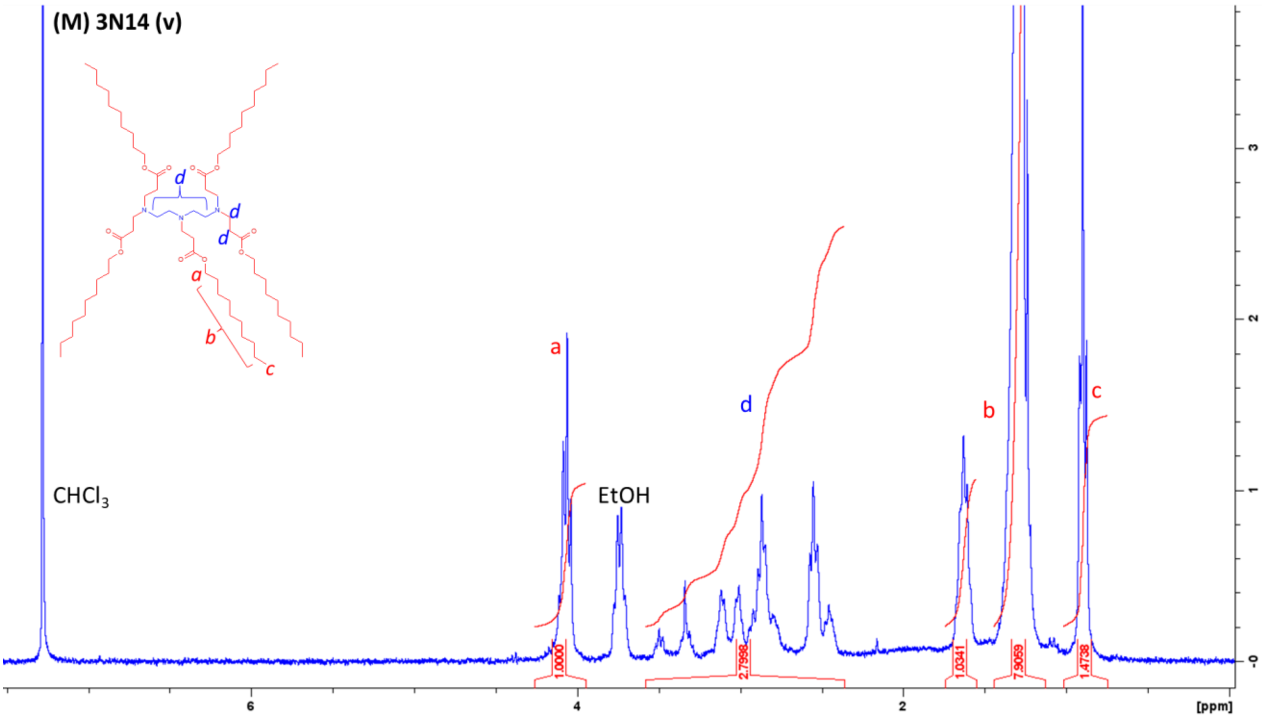

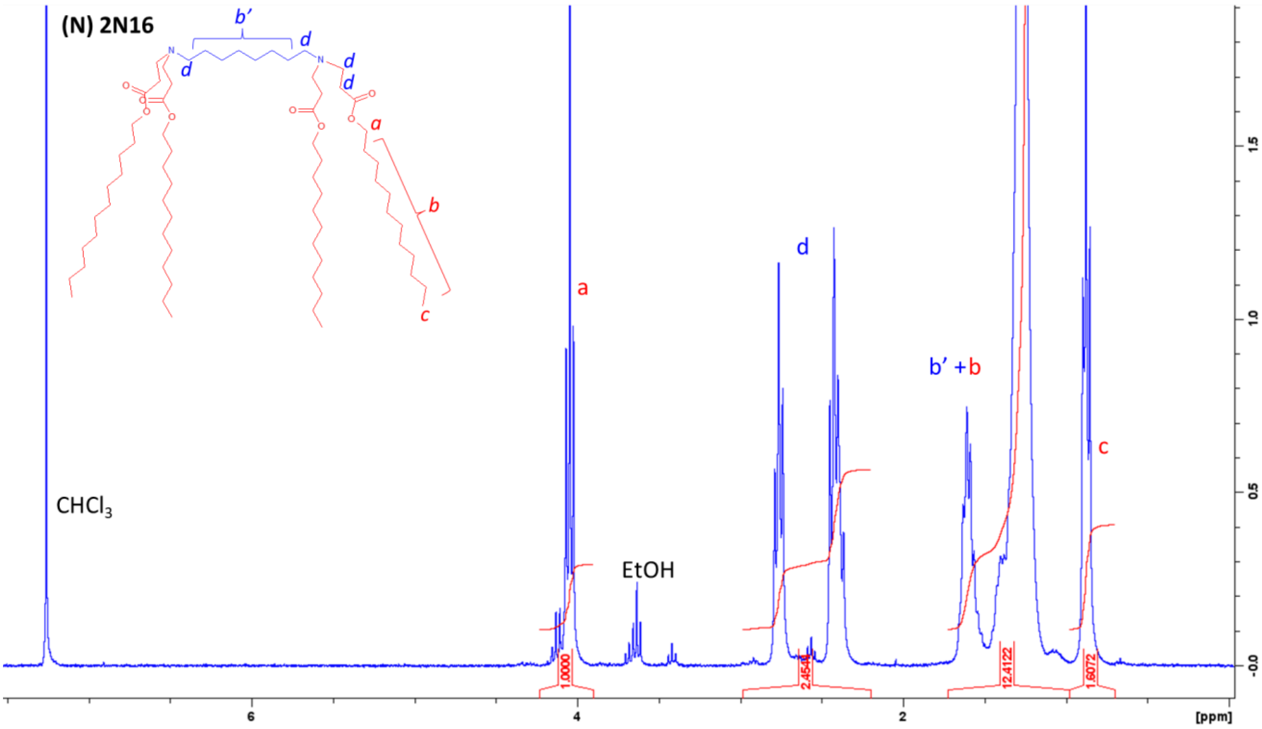

Figure S1: Representative ^{1}H NMR plots of lipidoids with peaks assigned to key protons shown in the chemical structures: (A) **3N$_6$B**, (B) **4N'8**, (C) **3N8$_{CB}$**, (D) **4N9**, (E) **4N$_C$9**, (F) **3N'9$_C$**, (G) **4N'10**, (H) **3N$_B$10$_B$**, (I) **4N'10$_B$'**, (J) **3N$_B$'11**, (K) **3N$_B$'12**, (L) **3N'13$_B$**, (M) **3N14**, and (N) **2N16**. Samples analyzed after vacuum treatment are notified with a (v). Degree of substitution was calculated from the integration of peaks from headgroup (labelled d), and tail group protons adjacent to the ester (labelled a).

Table S2: Summary of the scoring criteria for 104 lipidoids studied in the initial MIC screen study.

**Score**	**Condition**
0	No inhibition at any studied concentration
1	Delayed growth at 100 µM only
2	Delayed growth > 150 mins at 100 µM only
3	No growth at 100 µM only
4	Delayed growth at 50 µM
5	No growth at 50 µM
6	Delayed or no growth at 25 µM

Table S3: Scores awarded to each lipidoid based on initial antimicrobial screen.

	**2N**	**3N**	**3N'**	**4N**	**4N'**	**$3N_B$**	**$3N_B$'**	**$4N_C$**	Tail total
**6_B**	0	5	6	5	6	6	6	4	38
**8**	2	5	6	6	6	6	6	3	40
**8_{CB}**	4	4	6	3	6	6	6	6	47
**9**	4	6	6	6	6	6	6	6	51
**9_C**	2	3	6	4	6	1	6	6	39
**10**	3	6	6	6	6	6	6	6	51
**10_B**	3	6	5	3	6	5	4	6	43
**10_B'**	1	6	6	6	6	6	5	6	48
**11**	4	6	6	6	6	5	4	6	49
**12**	4	4	6	3	5	2	5	6	42
**13_B**	2	2	3	3	3	0	0	6	25
**14**	0	0	0	3	3	0	0	6	15
**16**	0	0	0	0	0	0	0	6	12
Head total	29	53	62	54	66	49	54	73	

Table S4: Quantifying parameters used for each tail group.

	MW	c log *P**
**6_B**	128.2	2.02
**8**	128.2	2.39
**8_{CB}**	208.3	4.22
**9**	142.2	2.92
**9_C**	206.3	3.69
**10**	156.2	3.45
**10_B**	198.3	4.49
**10_B'**	184.3	4.33
**11**	170.3	3.98
**12**	184.3	4.5
**13_B**	212.3	5.39
**14**	212.3	5.57
**16**	240.4	6.64

*c log *P* values taken from ChemSpider,[1] which lists values predicted using ACD/Labs logP)

Table S5: Quantifying parameters used for each headgroup.

	N/MW# (charge density)	c log *P**
**2N**	0.0139	1.1
**3N**	0.0291	-1.87
**3N'**	0.0206	-0.84
**4N**	0.0274	-2.18
**4N'**	0.0198	-0.96
**$3N_B$**	0.0200	-0.97
**$3N_B$'**	0.0212	-1.01
**$4N_C$**	0.0274	-2.68

#Number of nitrogens divided by molecular weight of the headgroup, *c log *P* values taken from ChemSpider,[1] which lists values predicted using ACD/Labs logP)

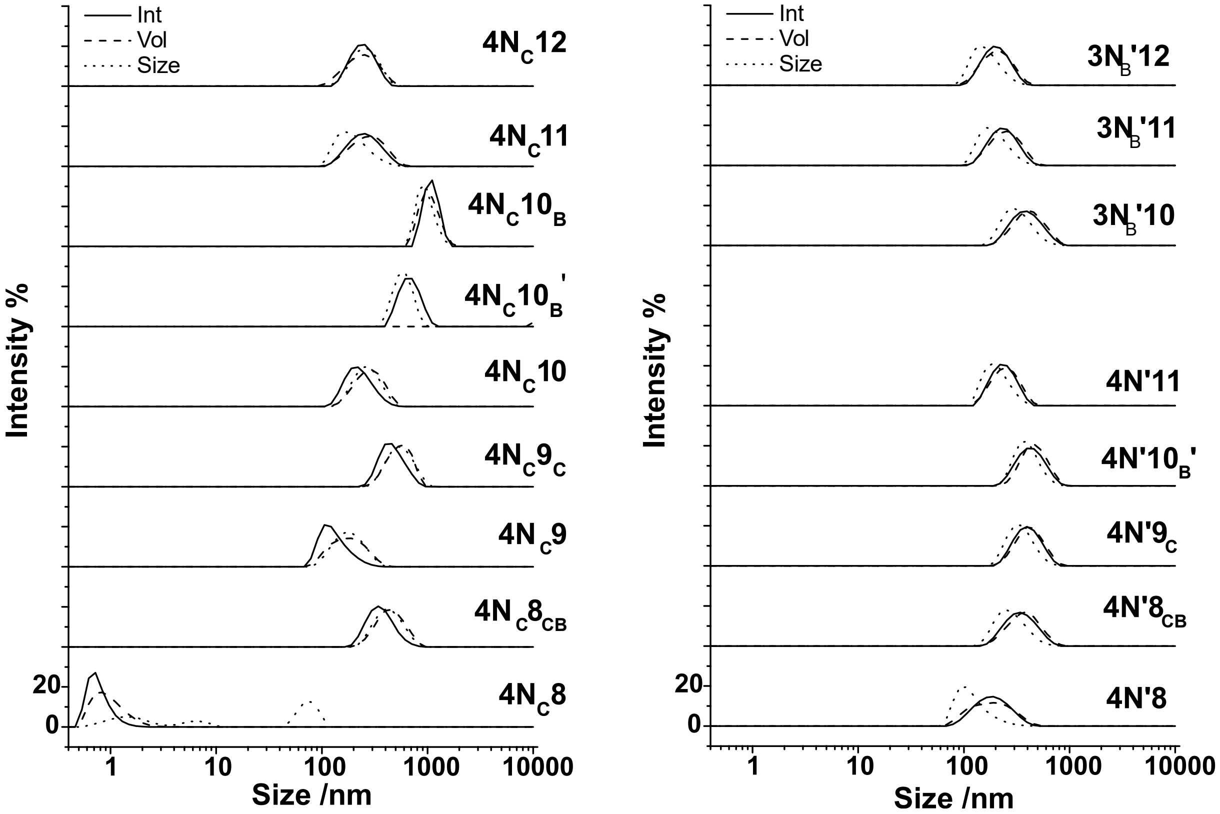

Figure S2: DLS data from various methylated lipidoids dispersed in LB medium at 2 µM. Three different distributions (intensity, volume, and size) are displayed in each case.

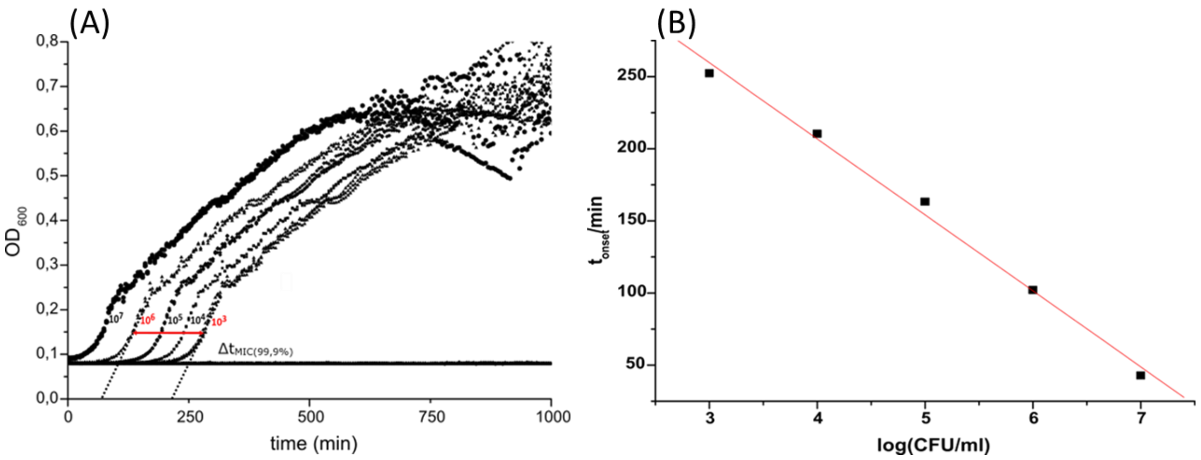

Figure S3: (A) Growth curves obtained for different cell densities (labelled) of *B. subtilis* in LB medium measured by bioscreen. Two linear regressions are shown for 10^3 and 10^6 CFU/ml, which were used to calculate (B) the onset of the log phase (t_{onset}) as the point at which the line intercepts the baseline OD value. From the time difference between t_{onset} at 10^6 and 10^3, the delay caused by a 99.9% reduction in cell growth was calculated from (B) as 150 mins. This value was then used as the criteria for measuring antimicrobial activity (Figure 1C) or MIC values (Table 1) in the growth assays.

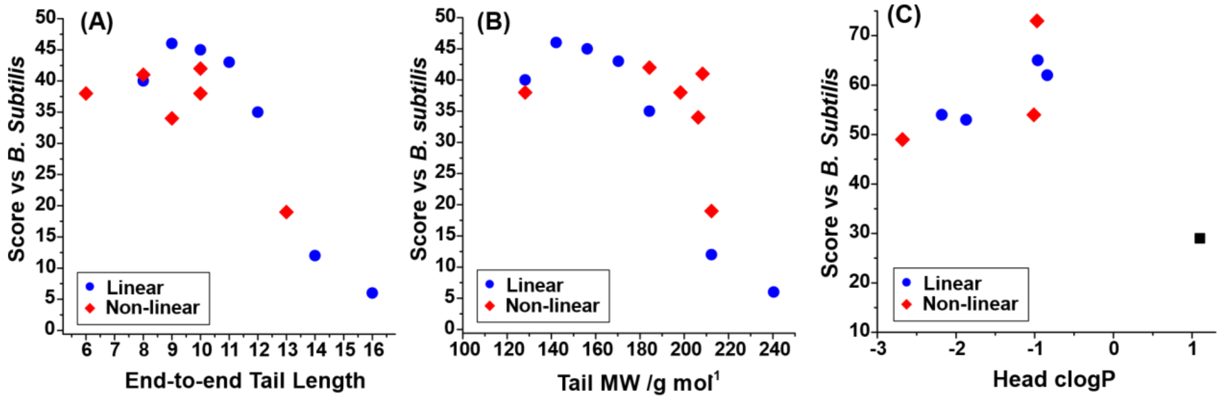

Figure S4: Additional structure-activity plots calculated based on the scores provided in Table S3 and the structural parameters listed in Tables S4 and S5. (A) Tail length end-to-end (i.e. the number in the code for each tail), (B) tail molecular weight, (C) headgroup c log *P* value. Lipidoid structures are grouped as linear or non-linear (tails and headgroups), and the gemini-like **2N** is also categorized separately (black square in C).

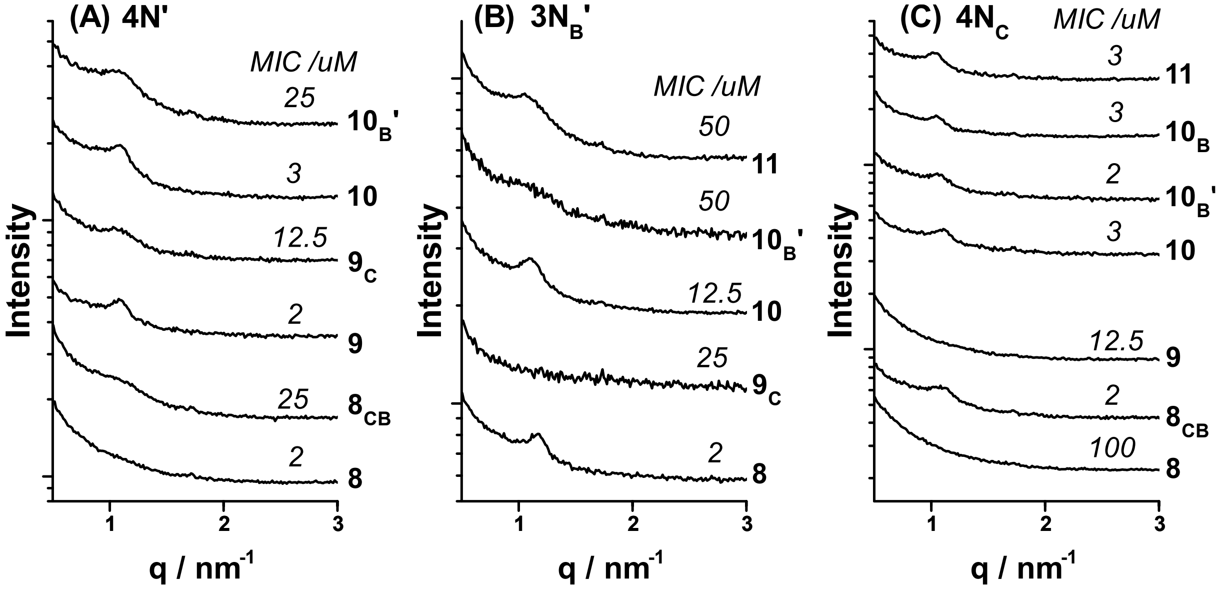

Figure S5: Additional lab source SAXS data for dispersed methylated lipidoids with (A) **4N'**, (B) **$3N_B$'** and (C) **$4N_C$** headgroups. Each is labelled with the MIC value for the given lipidoid against *B. subtilis*. A strong correlation between the most active lipidoids (MIC < 6 μM) and the appearance of structure within particles is evident.

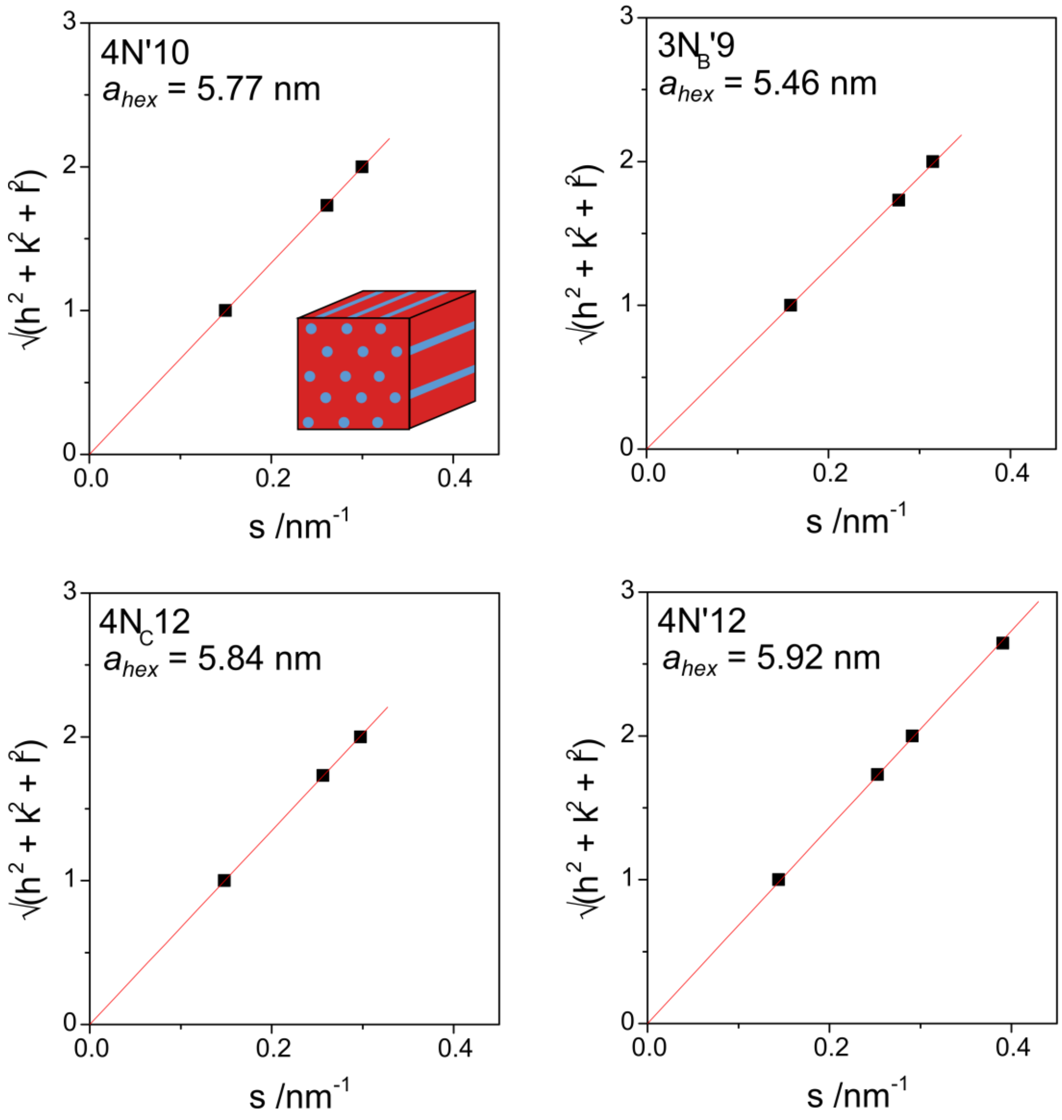

Figure S6: Indexing the hexagonal phase using peak positions from synchrotron SAXS data for four lipidoids (data in **Figure 2** and **S7**). Unit cell sizes (a_{hex}) were calculated from the inverse of the gradient of the linear fit, and are included on each plot.

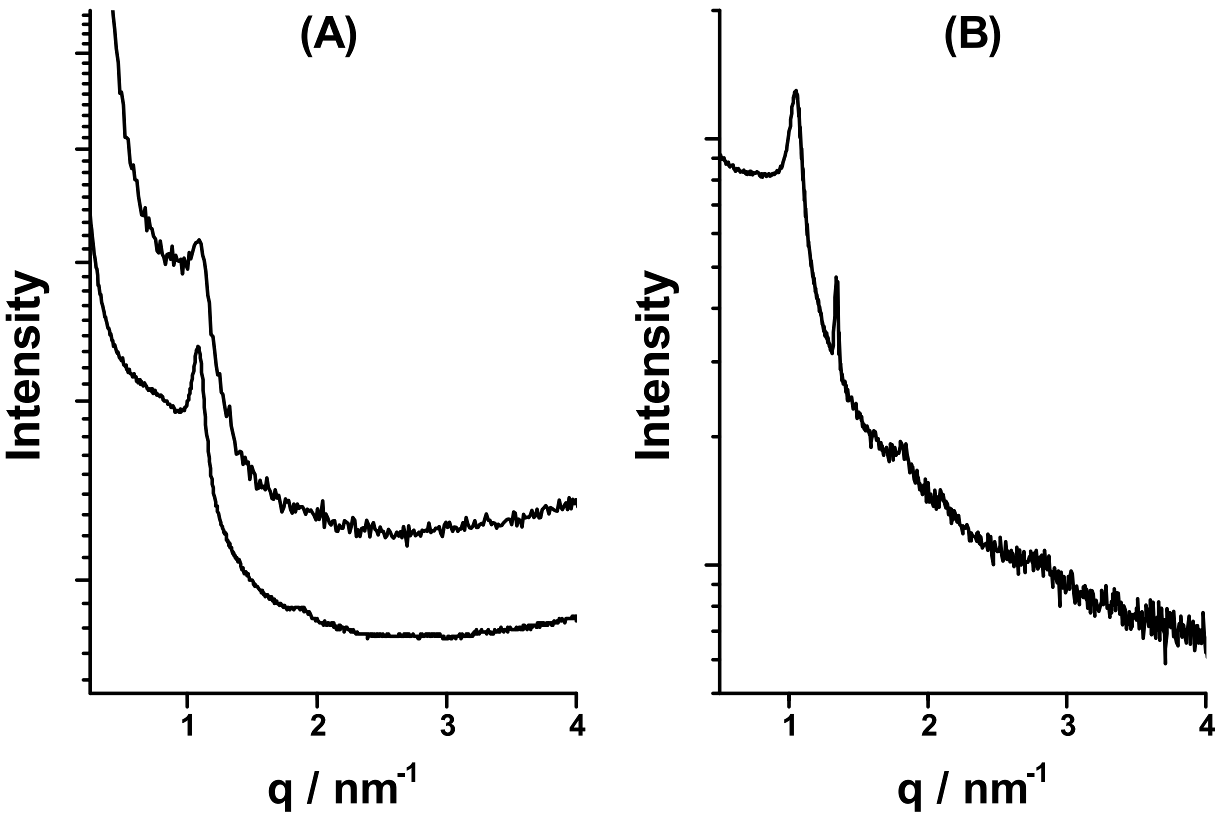

Figure S7: (A) Comparison of synchrotron (10 x 0.1 s exposure) and lab source (6 x 600 s exposure) SAXS data for **4N'10** and (B) Synchrotron SAXS data for **4N'12**, which showed hexagonal structure despite its relatively low antimicrobial activity (MIC = 50 μM). The sharp peak at ca. 1.3 nm^{-1} could not be assigned to a hexagonal phase or any other phase in combination with the other observed peaks.

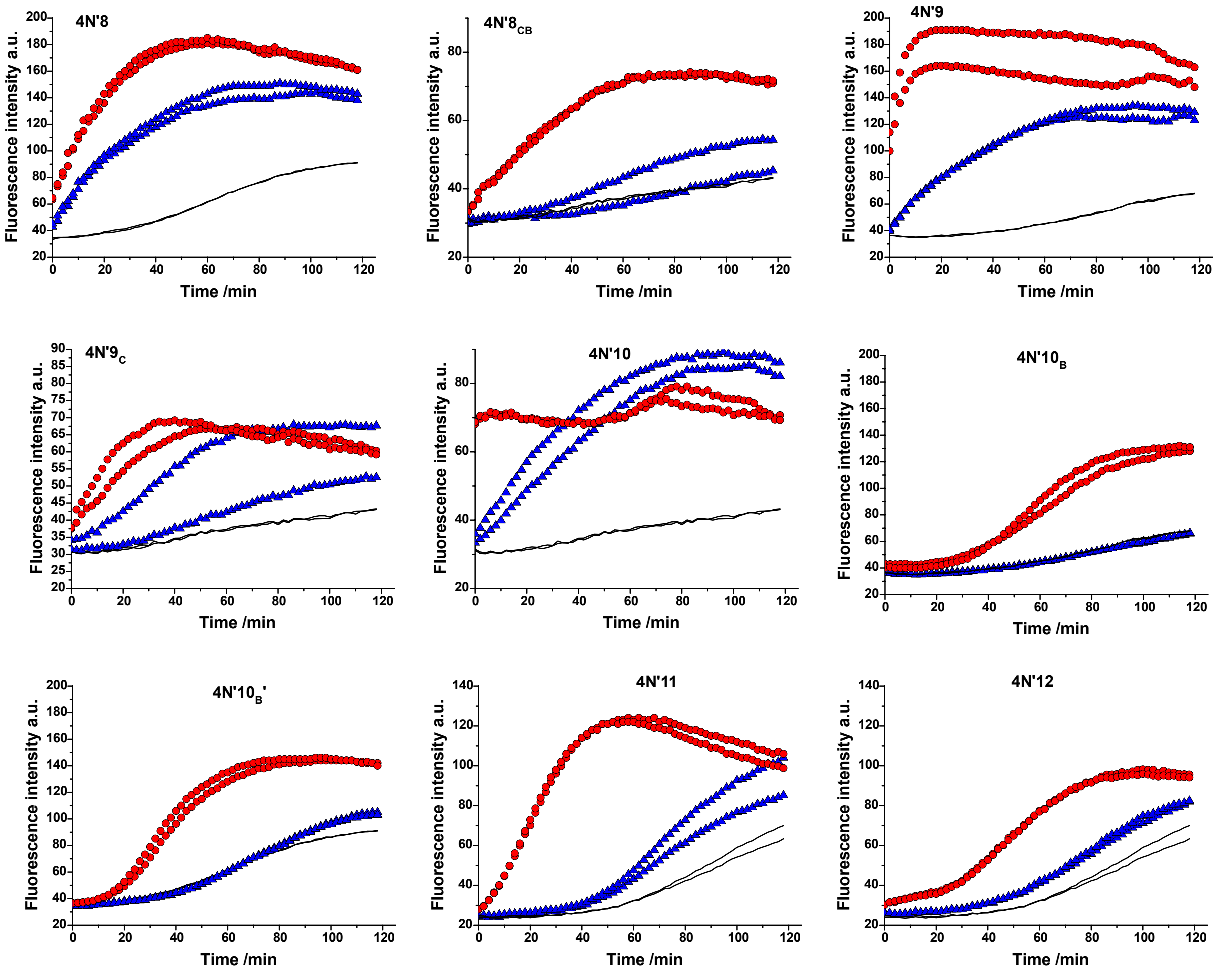

91F

Figure S8: Raw PI data from lipidoids with **4N’** headgroups at 25 μM (red) and 6.25 μM (blue), relative to control experiments (black).

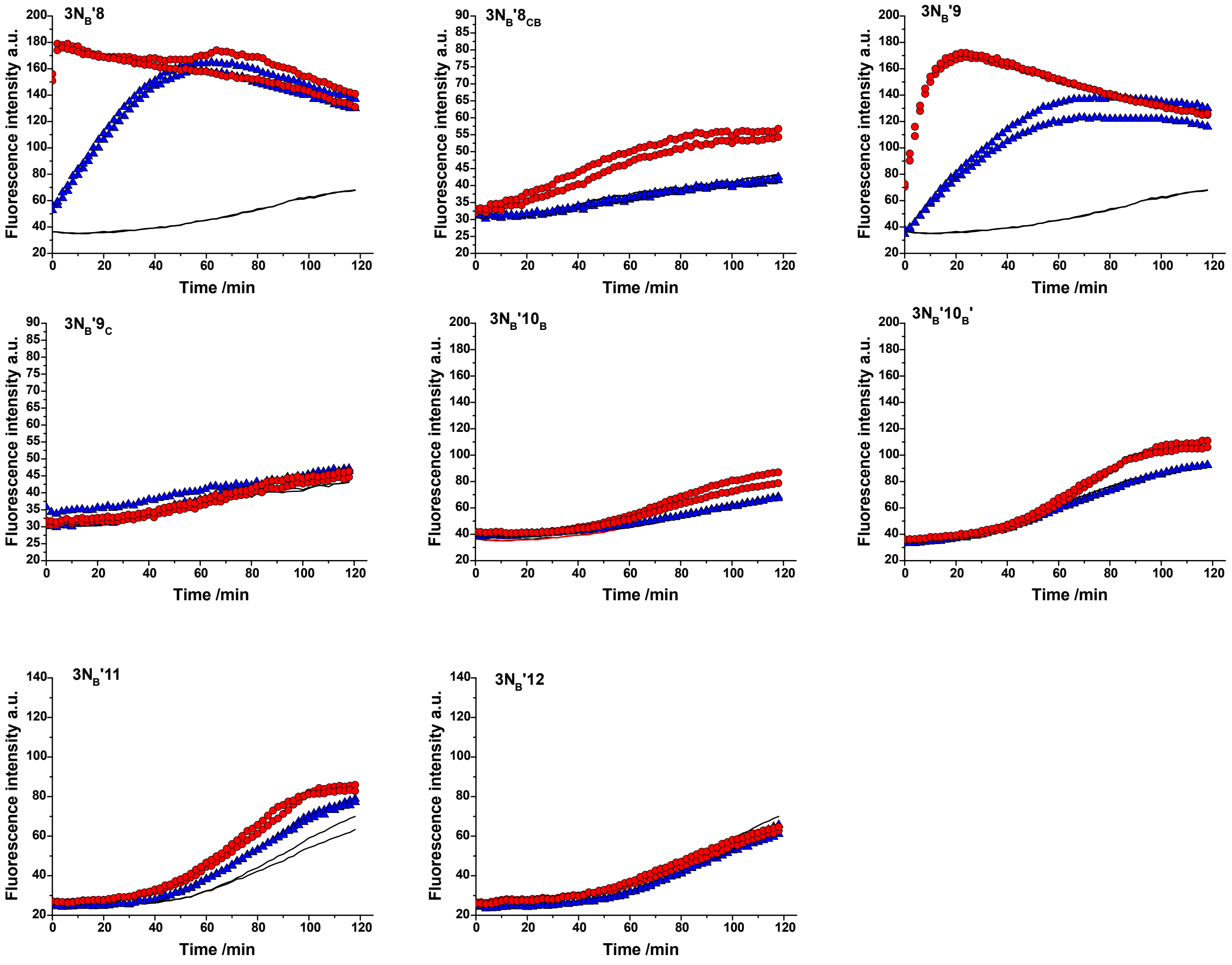

Figure S9: Raw PI data from lipidoids with **3N$_B$’** headgroups at 25 μM (red) and 6.25 μM (blue), relative to control experiments (black)

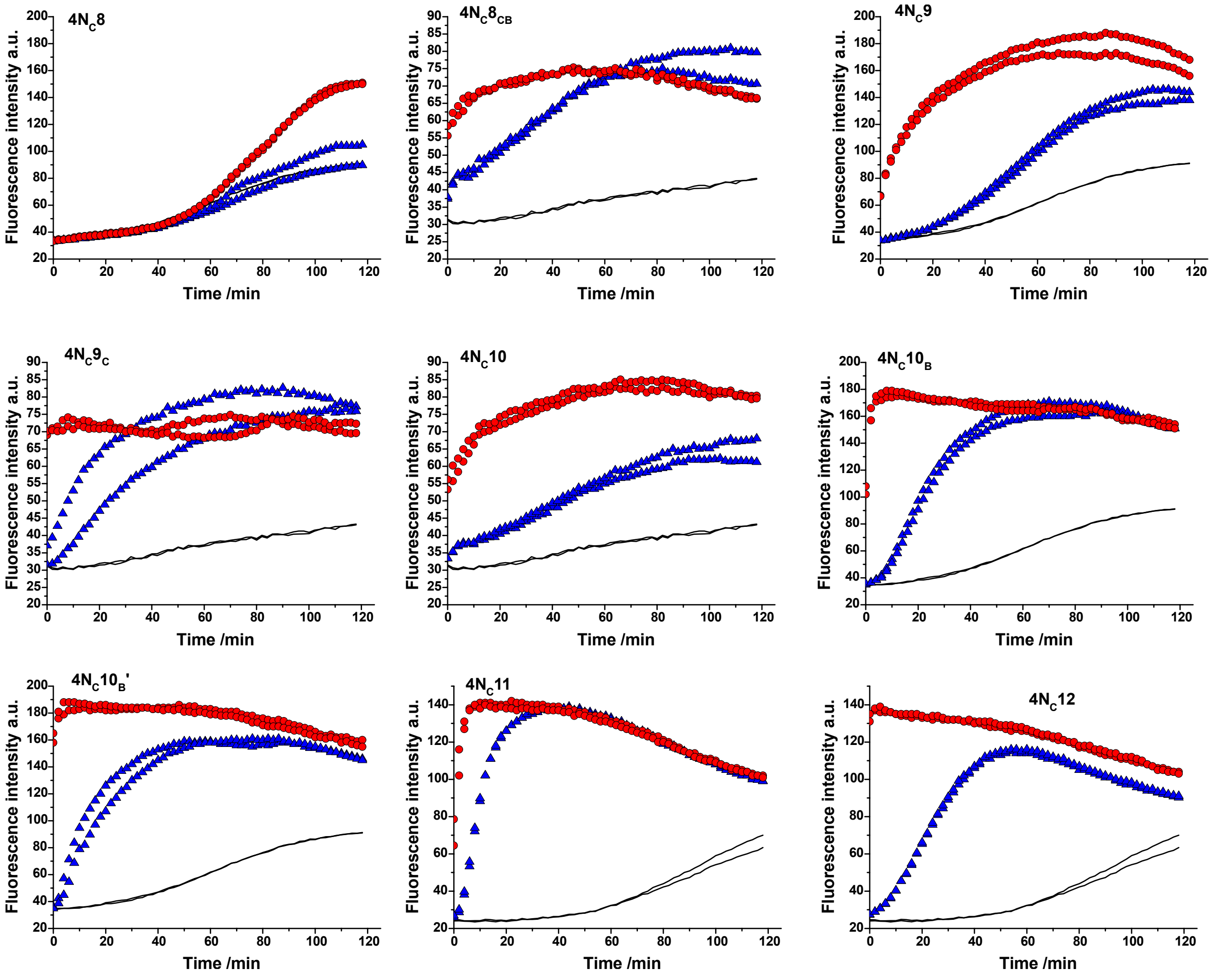

Figure S10: Raw PI data from lipidoids with **4N**$_{C}$ headgroups at 25 μM (red) and 6.25 μM (blue), relative to control experiments (black).

1 H. E. Pence and A. Williams, *J. Chem. Educ.*, 2010, **87**, 1123–1124.
